# Release of Small RNA-containing Exosome-like Vesicles from the Human Filarial Parasite *Brugia malayi*

**DOI:** 10.1371/journal.pntd.0004069

**Published:** 2015-09-24

**Authors:** Mostafa Zamanian, Lisa M Fraser, Prince N Agbedanu, Hiruni Harischandra, Andrew R Moorhead, Tim A Day, Lyric C Bartholomay, Michael J Kimber

**Affiliations:** 1 Department of Biomedical Sciences, Iowa State University, Ames, Iowa, United States of America; 2 Department of Molecular Biosciences, Northwestern University, Evanston, Illinois, United States of America; 3 Department of Infectious Diseases, University of Georgia, Athens, Georgia, United States of America; 4 Department of Pathobiological Sciences, University of Wisconsin-Madison, Madison, Wisconsin, United States of America; Institute of Medical Microbiology, Immunology and Parasitology, GERMANY

## Abstract

Lymphatic filariasis (LF) is a socio-economically devastating mosquito-borne Neglected Tropical Disease caused by parasitic filarial nematodes. The interaction between the parasite and host, both mosquito and human, during infection, development and persistence is dynamic and delicately balanced. Manipulation of this interface to the detriment of the parasite is a promising potential avenue to develop disease therapies but is prevented by our very limited understanding of the host-parasite relationship. Exosomes are bioactive small vesicles (30–120 nm) secreted by a wide range of cell types and involved in a wide range of physiological processes. Here, we report the identification and partial characterization of exosome-like vesicles (ELVs) released from the infective L3 stage of the human filarial parasite *Brugia malayi*. Exosome-like vesicles were isolated from parasites in culture media and electron microscopy and nanoparticle tracking analysis were used to confirm that vesicles produced by juvenile *B. malayi* are exosome-like based on size and morphology. We show that loss of parasite viability correlates with a time-dependent decay in vesicle size specificity and rate of release. The protein cargo of these vesicles is shown to include common exosomal protein markers and putative effector proteins. These *Brugia*-derived vesicles contain small RNA species that include microRNAs with host homology, suggesting a potential role in host manipulation. Confocal microscopy shows J774A.1, a murine macrophage cell line, internalize purified ELVs, and we demonstrate that these ELVs effectively stimulate a classically activated macrophage phenotype in J774A.1. To our knowledge, this is the first report of exosome-like vesicle release by a human parasitic nematode and our data suggest a novel mechanism by which human parasitic nematodes may actively direct the host responses to infection. Further interrogation of the makeup and function of these bioactive vesicles could seed new therapeutic strategies and unearth stage-specific diagnostic biomarkers.

## Author Summary

Lymphatic filariasis is caused by parasitic nematodes that invade and occupy the host lymphatic system. The extent of lymphatic filariasis is staggering, with over 120 million people infected in 73 endemic countries and an estimated 40 million people suffering from a range of disfiguring and debilitating clinical manifestations of this disease. The mechanisms by which these medically important parasites navigate the host immune response to establish infection are not yet fully understood. In this study, we identify exosome-like vesicles (ELVs) that are abundantly released from infective stage L3 *Brugia malayi*, an etiological agent of human lymphatic filariasis. We show that these vesicles have a narrow size distribution and morphology consistent with classical exosomes, and that they contain common exosomal protein markers, putative effector proteins, as well as small regulatory RNAs. We show that ELVs are enriched with microRNAs that are perfectly conserved between parasite and host, suggesting a potentially novel mechanism by which filarial worms can actively manipulate host gene expression. We demonstrate that parasite ELVs are internalized by macrophages and elicit a classically activated phenotype in these host cells. The discovery of exosome-like vesicle release by human nematode parasites newly enlightens the roadmap to understanding the pathology of LF and related helminthiases. These vesicles also present promising new targets for intervention and diagnostics.

## Introduction

The parasitic filarial nematodes *Wuchereria bancrofti*, *Brugia malayi* and *B. timori* are etiological agents of Lymphatic filariasis (LF), a chronic and debilitating disease infecting over 120 million people in 73 endemic countries [1]. Adult parasites reside in the lymphatic vasculature of infected individuals and release larvae called microfilariae, which are taken up by vector mosquitoes during the blood meal. Parasites rapidly develop within the mosquito, molting twice to the infective L3 stage [2, 3] before transmission to the definitive host during a subsequent blood meal. Following penetration of the vertebrate host via the puncture wound left by the mosquito, L3 stage parasites migrate to the lymphatics and undergo further growth and development, molting to the L4 stage and again to adulthood. The longevity of patent infection is remarkable; adults live for at least 8–10 years by general consensus. The ability of larval stages to successfully invade the host, and for adult worms to maintain infection for such an extended period of time, suggest filarial worms have developed strategies to both facilitate the establishment of infection and evade or manipulate the host immune response. Although the immunomodulatory capabilities of infecting larval and adult stage filarial worms have been well documented and reviewed [4–8], the parasite effector molecules responsible for manipulating host biology and their mechanisms of release have been difficult to define. Actively secreted proteins have historically been considered the principal candidates and several secreted proteins have been identified with demonstrable bioactivity at the host-parasite interface [9–12]. Adding to these, the characterization of parasitic nematode secretomes has revealed a complex array of potential proteinaceous effectors [13–16]. Other types of effector, including molecules expressed on the parasite surface may have a role [17] and the emergence of small noncoding RNAs as cell-to-cell agents of genetic regulation [18–22] hint at exciting alternative mechanisms.

Exosomes are a subtype of extracellular vesicle categorized by size (30–120 nm diameter) and defined by a particular biogenic pathway [23]; exosomes are formed by inward budding of vesicles in the late endosomal pathway to create multivesicular endosomes that fuse with the plasma membrane to effect release [24, 25]. Originally thought to be a means of cellular waste disposal, exosomes are now considered highly bioactive extracellular vesicles that facilitate cell-to-cell communication and are the focus of renewed investigation. The cargo of exosomes is complex and variable, containing bioactive proteins, functional mRNA, miRNA and other small non-coding RNA species [18, 26], likely reflecting both source and target environments. Fusion of the exosome to a target cell delivers this heterogeneous bioactive cargo and selectively alters the biology of the target tissue [19, 21, 26, 27]; the isolation of exosomes from circulatory systems and an array of biofluids suggests effector sites can be far from the point of release. Parasites are known to release exosome-like vesicles [27–30] and it is compelling to hypothesize that bioactive molecules secreted by parasitic nematodes, packaged in exosomes, function as cell-to-cell effectors in the host-parasite interaction. Indeed recently, extracellular vesicles secreted by the gastrointestinal nematode *Heligmosomoides polygyrus*, containing proteins and small RNA species, have been shown to alter gene expression in host cells and suppress innate immune responses in mice [26].

Here we show that larval and adult stage *B. malayi* secrete prodigious quantities of extracellular vesicles *in vitro* whose size and morphology are consistent with exosomes. These exosome-like extracellular vesicles (ELVs) contain small RNA species, including specific miRNA and are enriched in miRNA that are identical to host miRNAs with known immunomodulatory roles [31–34]. The protein cargo of the vesicles is relatively scant but includes bioactive proteins, proteins with putative RNA binding properties and proteins commonly associated with exosomes [35]. The parasite ELVs are internalized by host macrophages and elicit a classically activated phenotype in these cells. The demonstration that filarial nematodes secrete exosomal RNA and proteins that potentially function at the host-parasite interface is significant. Defining this parasite effector toolkit exposes an array of new molecules that may be exploited in novel LF control strategies.

## Results and Discussion

### Infective-stage *B. malayi* release exosome-like vesicles

**Fig 1:**
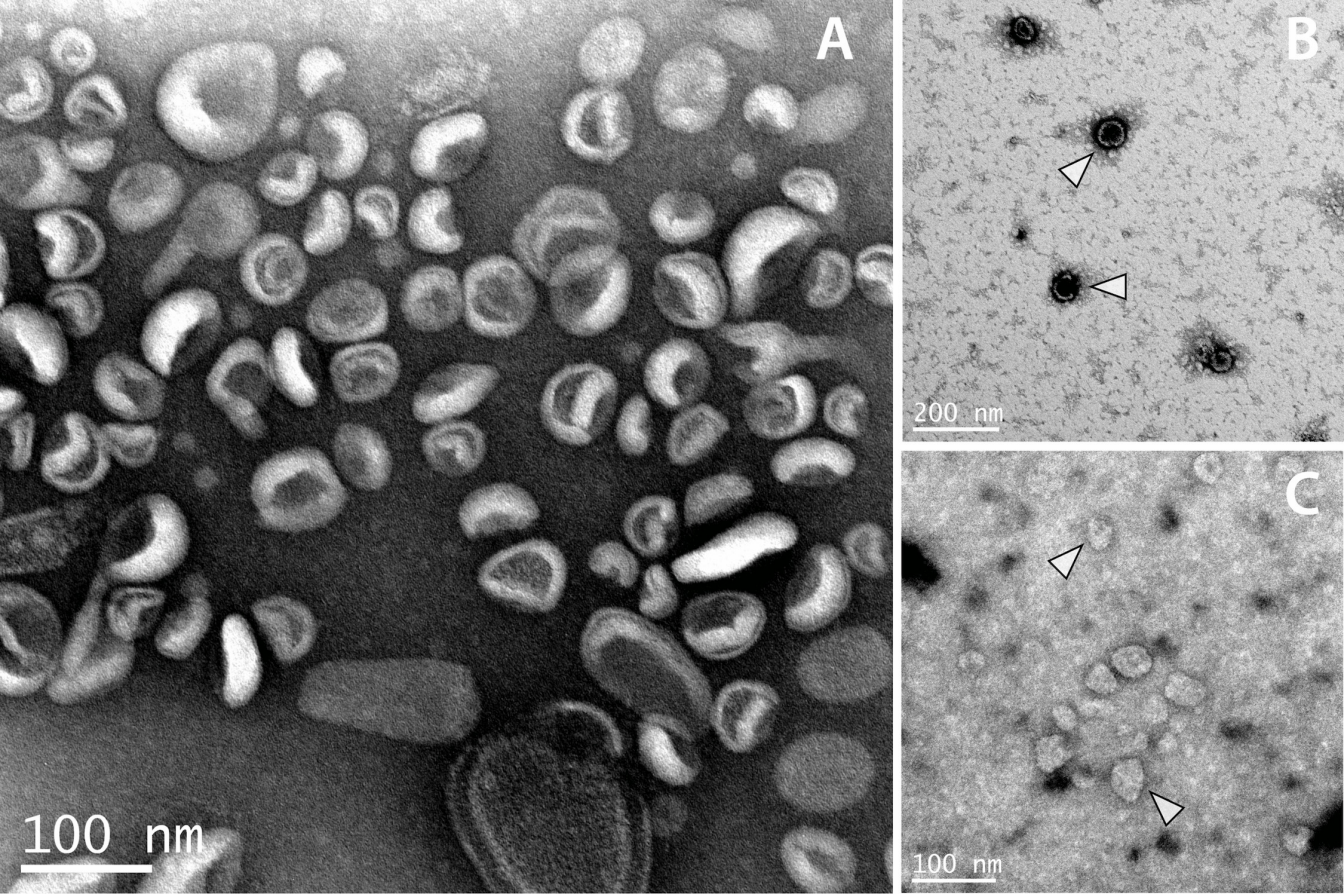
Electron microscopy confirms secretion of exosome-like vesicles in intra-host stages of *B. malayi*. TEM images of L3 (A and B) and adult female (C) ELV preparations are shown. L3 vesicles take on a distinct morphology often reported in the literature. Adult isolations are more heterogenous and may require further optimization to achieve uniform vesicle preparation. White arrows show canonical L3 ELVs (B) and putative adult ELVs (C). This provides evidence for the release of exosome-like vesicles in the human-infective L3 stage of the parasite and much of the rest of the work we report is focused on vesicles derived from this larval stage.

In order to ascertain whether exosomes are released by *B. malayi*, extracellular vesicles were isolated from parasites incubated in culture media using a filtration and ultracentrifugation protocol. We focused our initial discovery efforts on larval and adult stage parasites. L3, adult male, and adult female *B. malayi* were incubated *in vitro* for 24 hour periods under standard culture conditions, and purified vesicle preparations were evaluated with electron microscopy (EM). Infectious stage L3 parasites in culture release abundant 50–120 nm microvesicles consistent with the classical “deflated ball” morphology of mammalian and non-mammalian exosomes reported in the literature [36] (Fig 1A & 1B). We refer to these as exosome-like vesicles (ELVs) throughout this manuscript, in recognition that they cannot be unequivocally designated as exosomes, rather than another class of extracellular vesicles, because their biogenesis has not been determined. Preparations from adult stage *B. malayi* were more heterogenous and dilute, not allowing for the definitive categorization of putative exosome-like vesicles (Fig 1C). This, despite the fact a much higher mass of total parasite tissue was used for adult preparations as compared to larval preparations. These data suggest ELV release to be a predominantly larval phenomenon in *B. malayi*, a working hypothesis supported by analysis of RNA associated with the vesicles. We therefore chose to focus our subsequent experiments on L3 stage parasites. A compelling overall hypothesis for the function of *B. malayi* ELVs is that they mediate the secretion and trafficking to host cells of effector molecules that facilitate parasitism and the observation that ELV secretion occurs primarily in those parasite stages that infect the host and establish parasitemia is consistent with this narrative.

### Time course profile of exosome-like vesicle release from infectious stage *B. malayi*

**Fig 2:**
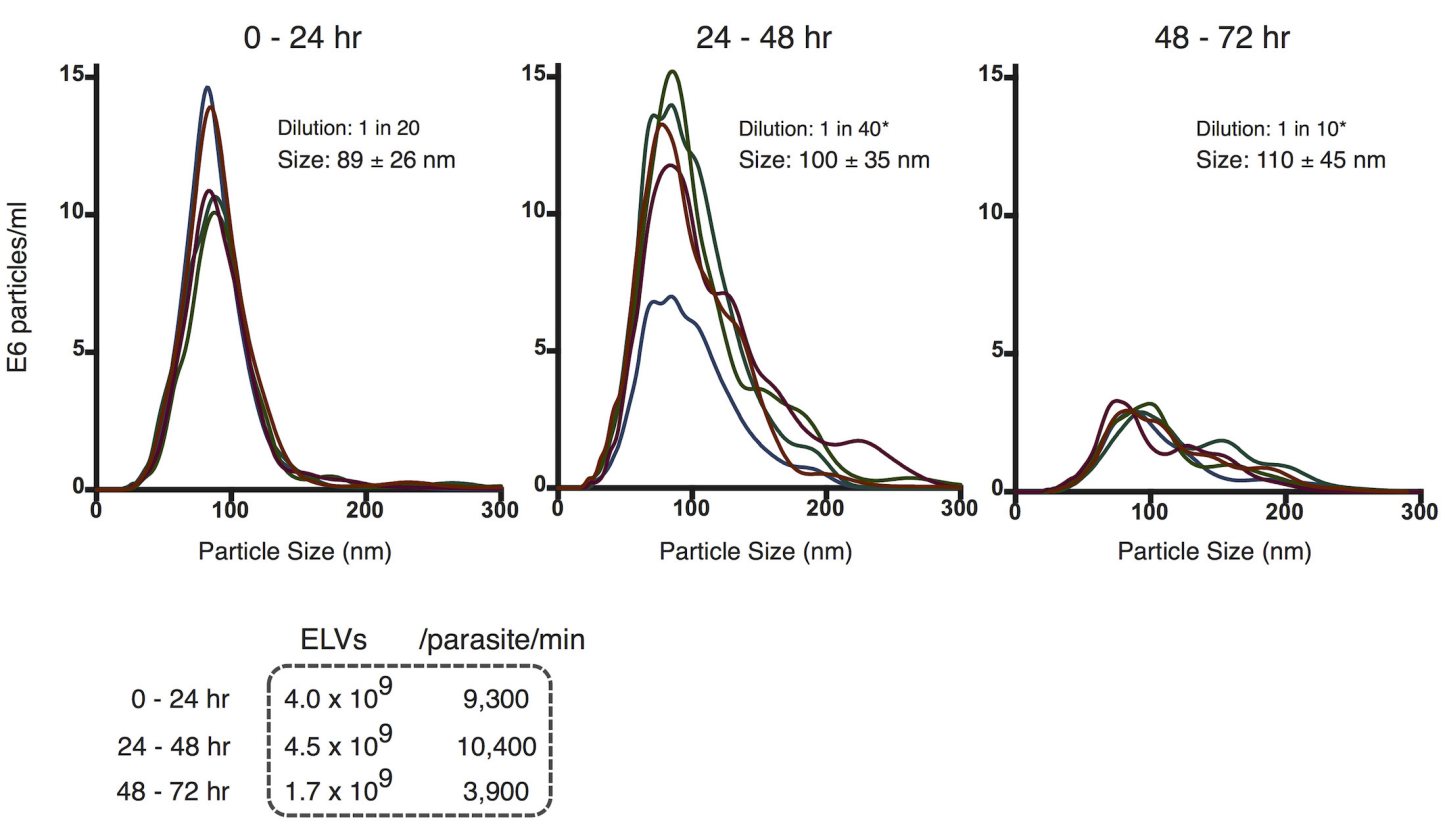
Particle tracking analysis reveals prolific larval *Brugia* exosome-like vesicle release rate. Profile of ELVs isolated from culture media incubated with 300 L3 parasites for successive 24 hr incubations. The size distribution of L3-derived ELVs from Day 1 (left), Day 2 (center) and Day 3 (right) incubations are shown (mean ± SD). Calculated vesicle release rates are provided in tabular format. ELV rate of release and size specificity decay in a time-dependent manner *in vitro*. * re-scaled based on dilution for comparison to 0–24 hour (1:20) dilution.

To more accurately resolve the dynamics of ELV release in L3 *B. malayi*, we used a nanoparticle tracking analysis (NTA) system to measure vesicle output in a 72 hr *in vitro* time course. Media was collected from 300 worms after three successive 24 hr incubation periods, vesicles were purified by ultracentrifugation as before and individual vesicle preparations were analyzed via NanoSight LM10 as shown in Fig 2 (sample recording: S1 Video). Day 1 (0–24 hr in culture) preparations reveal a prolific ELV release rate (> 9,000 ELVs/parasite/min) with a very narrow size distribution centered at ∼90 nm. Day 2 (24–48 hr in culture) preparations show an essentially equivalent rate of release, but a stark broadening of the size distribution. Day 3 (48–72 hr in culture) preparations are associated with significantly lower levels of release (<4,000 ELVs/parasite/min) and an even wider multimodal size distribution. These data suggest an overall time-dependent decay in vesicle rate of release and size specificity, which correlates to decreased L3 viability *in vitro*. The release of considerable quantities of precisely-sized ELVs in viable worms (Days 1–2) is followed by the release of smaller quantities of a broader size range of particles that potentially include larger membrane vesicles and apoptotic blebs (Days 2–3). This suggests an active and regulated mechanism of ELV release in healthy and viable L3 stage parasites, as opposed to a passive mode of noisy cellular deterioration.

### The protein cargo of *Brugia* exosome-like vesicles

The protein content of *B. malayi* ELVs was determined using nanoscale liquid chromatography coupled to tandem mass spectrometry (nano LC/MS/MS). A total of 32 proteins each containing at least two unique peptides were identified using MASCOT (Table 1). Specific proteins identified within the pellet included characteristic markers of exosomes including Hsp70, elongation factor-1*α*, elongation factor-2, actin, and Rab-1. In addition, over 80% of the proteins identified are orthologous to proteins identified in mammalian exosome proteomes, strongly suggesting that these vesicles are exosome-like in nature and supporting our ELV designation here. Interestingly, this set of vesicle-specific proteins is entirely distinct from the proteins previously identified in pre- and post-molt L3 secretions [37].

**Table 1:** Annotation of *Brugia* ELV proteome.

*Brugia* **Protein ID**	**UniProt ID**	**Annotation**
BM-ACT-5	A8P5A0_BRUMA	Actin
Bm-HSP-1	A8P6Q6_BRUMA	HSP70
Bm5195	A8PJ17_BRUMA	Elongation factor 1-alpha
BM-EEF-2	A8PJV1_BRUMA	Elongation factor 2
Bm4733	A8PHP7_BRUMA	Beta-tubulin
BM-MEC-12	A8PN52_BRUMA	Alpha-tubulin like
BM-DPY-23	A8PZJ6_BRUMA	Adaptin
Bm13837	A8QAR6_BRUMA	ATP synthase subunit alpha
Bm-ATP-2	A8Q895_BRUMA	ATP synthase subunit beta
Bm5931	A8P4C6_BRUMA	Alpha-1,4 glucan phosphorylase
BM-CPL-1	A8NCV6_BRUMA	Cathepsin L-like cysteine protease
BM-EAT-6	A8Q4C9_BRUMA	Na^+^K^+^ATPase
Bm-SCA-1	A8QET1_BRUMA	Calcium-transporting ATPase
BM-RAB-1	A8Q8U0_BRUMA	Ras-related protein
Bm4628	A8NSV0_BRUMA	Ubiquitin
Bm5528	A8NHQ1_BRUMA	1,4-alpha-glucan branching enzyme
BM-DLST-1	A8PU77_BRUMA	2-oxoglutarate dehydrogenase
Bm3206	A8NHD8_BRUMA	Histone H2B
Bm3425	A8NHD2_BRUMA	Histone H3
Bm4113	A8Q1K1_BRUMA	Histone H4
**Ribosomal Proteins**		
BM-RPS-16	A8P1D4_BRUMA	40S ribosomal protein S16
Bm2853	A8Q0J1_BRUMA	40S ribosomal protein S2
Bm2320	A8NXR7_BRUMA	40S ribosomal protein S3
BM-SECS-1	A8PJH5_BRUMA	40S ribosomal protein S5
BM-RPS-9	A8P2X1_BRUMA	40S ribosomal protein S9
Bm13774	A8PTY7_BRUMA	60S ribosomal protein L10
Bm13718	A8NKQ0_BRUMA	60S ribosomal protein L11
BM-RPL-3	A8P136_BRUMA	60S ribosomal protein L3
BM-RPL-9	A8QHP9_BRUMA	60S ribosomal protein L9
Bm3930	A8ND23_BRUMA	Ribosomal protein
BM-RPL-1	A8NG31_BRUMA	Ribosomal protein

Homology-based annotation of *B. malayi* ELV proteins reveals hallmarks of mammalian exosomes, including HSP70 and translation elongation factors. Ribosomal proteins, histones, ras-related proteins, cathepsins, ATP synthase subunits, and other homologs of identified *Brugia* ELV proteins have also been reported in exosomes derived from various cell types.

**Fig 3:**
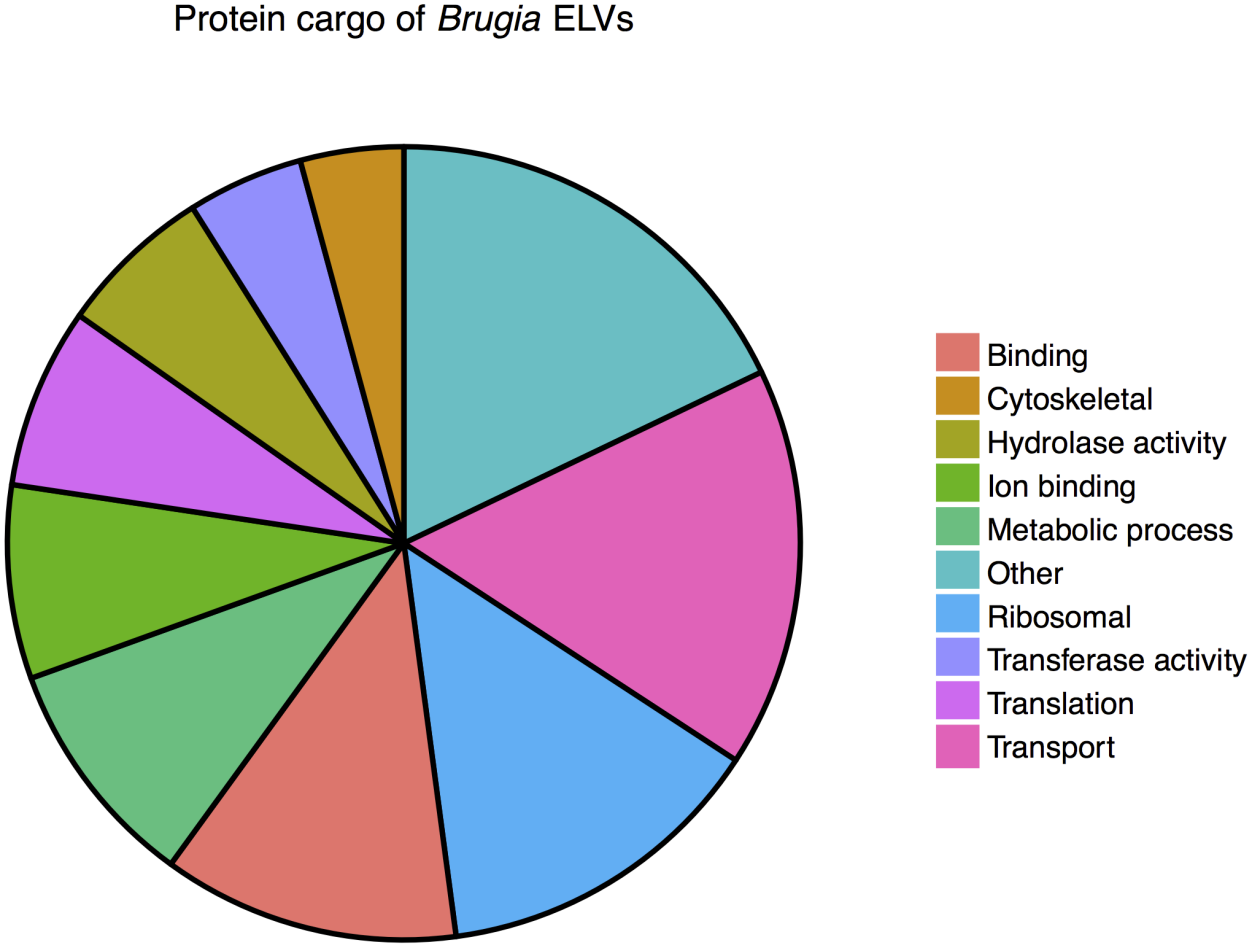
Protein content of *B. malayi* exosome-like vesicles. GO functional annotation of 32 proteins identified in ELVs isolated from *B. malayi* L3 stage parasites.

UniProt-GOA and quickGO were used to sort proteins into functional groups based on assigned gene ontology (GO) terms [38, 39], as shown in Fig 3. Based on GO annotations, 20% of the proteins identified are involved in binding of bioactive molecules including nucleic acids and other proteins, 16% function in the transport of various ions and proteins and 14% are ribosomal proteins. In addition, a large fraction of proteins identified (21%) appear to be involved in various metabolic processes including hydrolase and transferase activities while the remaining 29% comprises proteins with translational, cytoskeletal and other functions.

Included in the list of *Brugia* ELV proteins are potential effector molecules. Bm-CPL-1 is a cathepsin L-like cysteine protease robustly expressed across the *B. malayi* life cycle [40]. Upregulation of *Bm-cpl-1* expression coincides with transition between life cycle stages and an important role in the modulation of parasite molting has been confirmed [41–43]. This is the first demonstration that *B. malayi* secretes CPL-1 although other cathepsin-like cysteine proteases have been identified in the *B. malayi* secretome [14, 37] and a cathepsin L-like molecule is secreted by intra-mammalian stage *Haemonchus contortus* [44]. The exogenous function of exosomal Bm-CPL-1 is not clear but evidence points to some manipulation of the host-parasite interface. In a previous study, we suppressed *Bm-cpl-1* expression using *in vivo* RNAi during the mosquito life stages [42]. Loss-of-function reduced prevalence of infection in mosquitoes by nearly 40%, suggesting Bm-CPL-1 is important for establishing or maintaining parasitemia. In flatworms, an immunomodulatory role for secreted cathepsin L-like proteases is better established [45]; in *Fasciola* infection cathepsin L contributes to the permissive polarized Th2 > Th1 host response.

The proteomic profiles of parasitic helminth exosomes are broad in range; for example, over 350 proteins were identified in the putative exosomes secreted by *Heligmosomoides polygyrus* [26] whilst 45 and 79 proteins were identified in exosome-like vesicles from *Echinostoma caproni* and *Fasciola hepatica*, respectively [46]. The *B. malayi* L3 stage profile identified here is relatively scant but consistent with this broad distribution. It may be that this is a stage-specific observation and ELV secreted by other *B. malayi* life stages display a more complex and abundant protein cargo tailored to distinct functional demands. Reflecting the small RNA component of these ELVs (see later sections), it may also be that larval stage *Brugia* ELVs are primarily vehicles for protected RNA secretion. Replication of the experiments conducted here might add depth to the MS data set and identify further ELV-associated proteins.

### *B. malayi* ELVs contain small RNA including miRNAs with potential host targets

**Fig 4:**
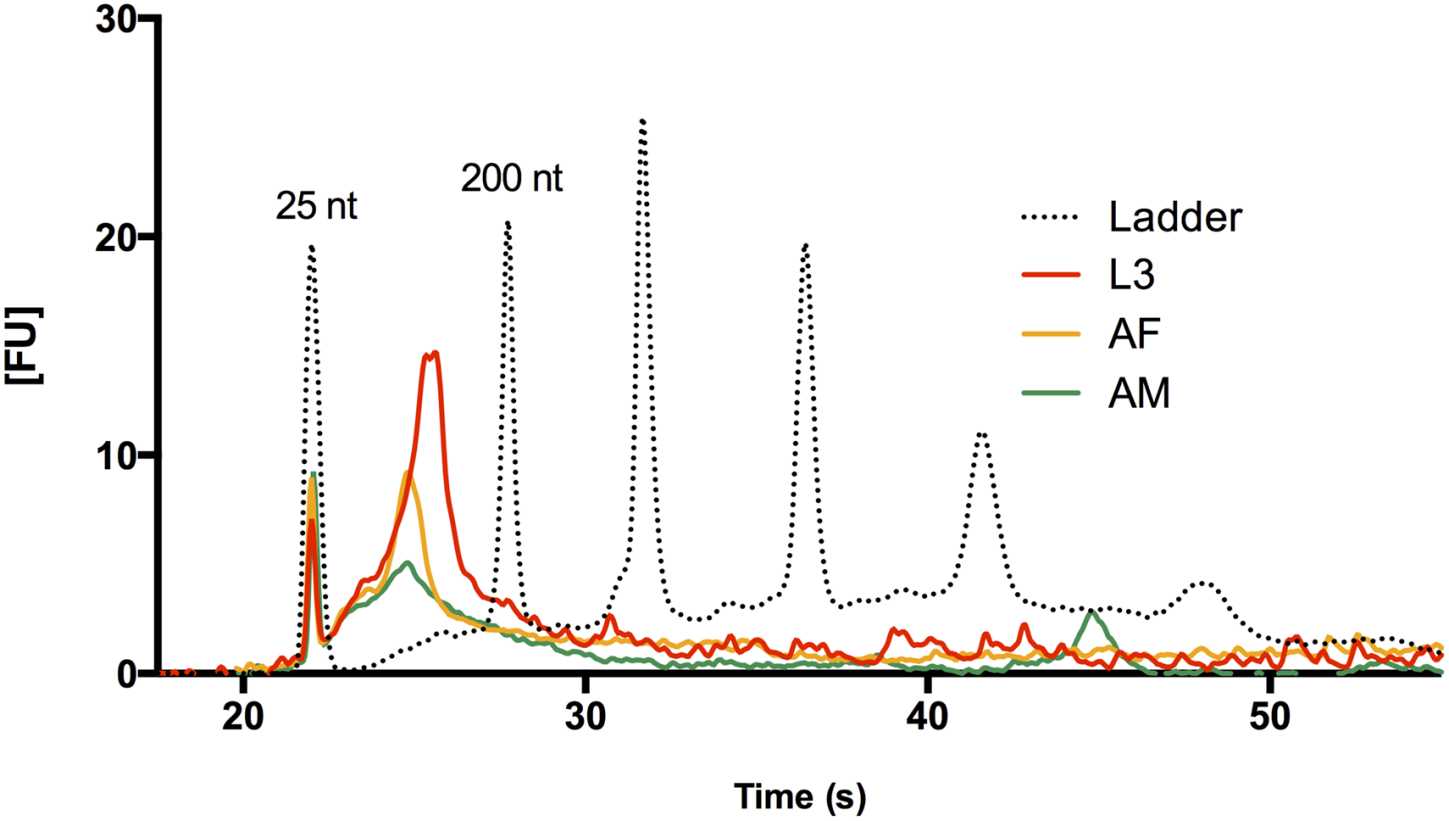
Isolation of Small RNAs from larval and adult *B. malayi* ELV fractions. Bioanalyzer data are shown for RNAs isolated from L3, adult male, and adult female *Brugia* preparations. L3 ELVs contain significant amounts of small RNAs in the 25–200 nt range (25 and 200 nt reference peaks labeled), while adult male and female vesicle preparations yield fewer RNAs. Vesicle fractions were prepared from 300 L3 and 30 adults in 24 hr culture incubations. Despite the much higher total tissue amounts used in adult culture, we detect much higher levels of small RNAs in L3-derived ELVs.

We probed larval and adult microvesicle preparations for the presence of small RNA species. Exosomes have been found to contain both non-coding RNAs (ncRNAs) and messenger RNAs (mRNAs) in a diverse range of species and cell types. Of particular interest to us was the potential presence of small non-coding RNAs, including microRNAs (miRNAs), that could potentially mediate parasite-parasite communication or modulate host gene expression. Small RNA species were preferentially isolated from putative ELV-containing pellets and examined with an Agilent Bioanalyzer. The microvesicle fractions of L3 *B. malayi* (24 hr incubations of 300 worms) revealed an abundance of small RNA species in the 25–200 nt range (Fig 4). Much less RNA was detected from incubations of adult male and female *B. malayi* (24 hr incubations of 30 adult worms), despite the much higher mass of tissue in adult stage culture media. This lack of correlation between total parasite tissue material and RNA yield, coupled to the differential quality of larval and adult microvesicle preparations as evaluated by EM, further indicates that ELV release is primarily a characteristic of larval-stage parasites and perhaps more biologically relevant to early parasite infection.

**Fig 5:**
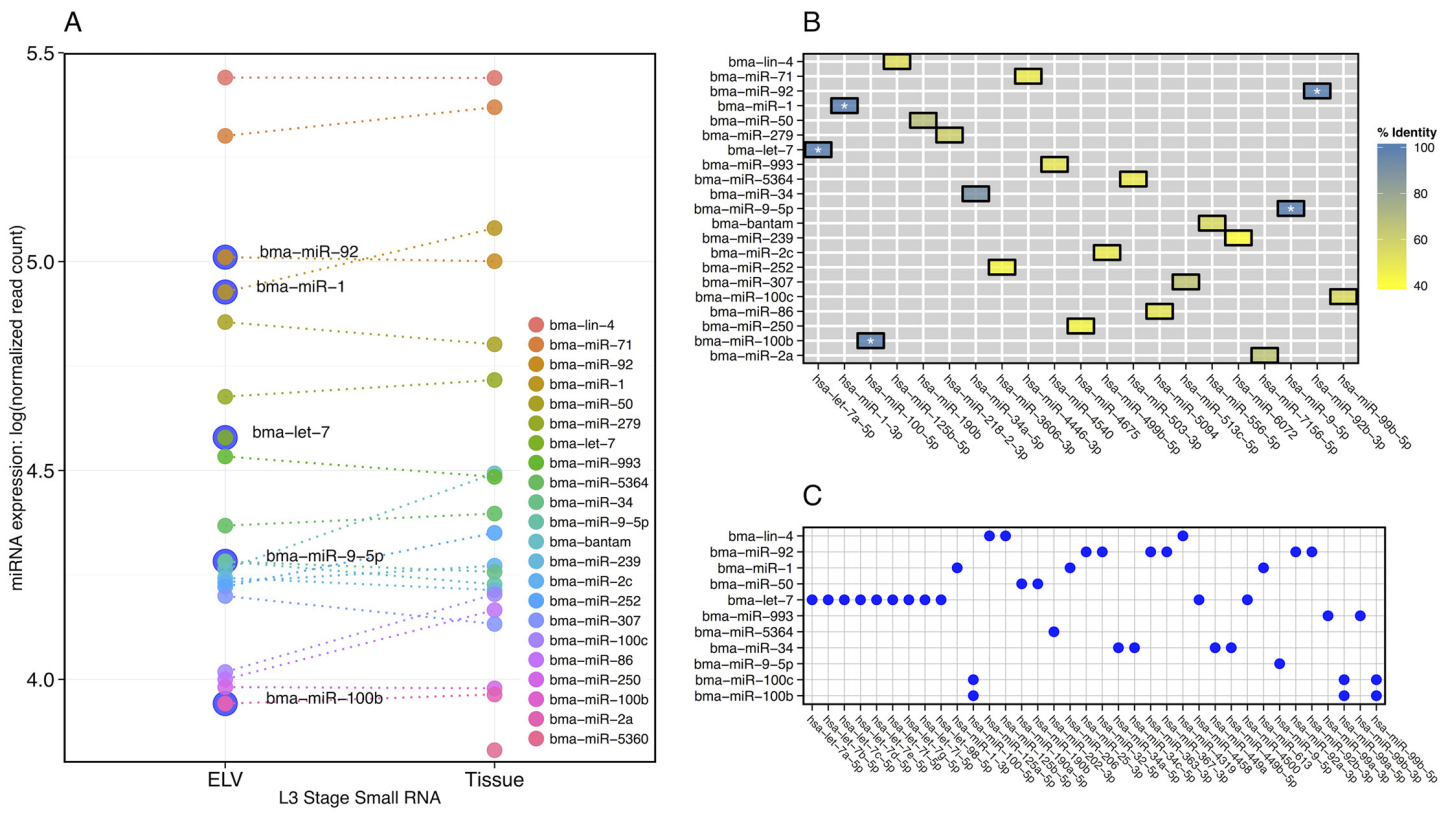
Discovery and profiling of miRNAs in *B. malayi* exosome-like vesicles. **(A) Comparative abundance of miRNAs in L3 ELV and tissue-derived samples**. miRNA discovery and abundance estimation was carried out using the mirDeep2 pipeline. The 20 miRNAs with highest expression in each sample were retained for comparison and abundance was normalized with respect to total miRNA-mapping reads within each sample. Normalized read count is plotted on a log scale for ELV and tissue miRNAs to provide a relative ordering of fractional abundance. Bma-let-7 only appears in the highly expressed subset, and a number of miRNAs with perfect mature sequence identity to host homologs are highlighted (outer blue circle). **(B) Sequence conservation between *B. malayi* ELV-origin miRNAs and the host ( *H. sapiens* ) miRNA complement**. Reduced heat map showing one-to-one homology between a given *B. malayi* miRNA and its nearest matching human counterpart in terms of percent identity. Bma-let-7, bma-miR-1, bma-miR-9, bma-miR-92, and bma-miR-100b (white asterisks) share 100% identity with a host miRNA, while bma-miR-34 shows high identity with a host miRNA (21/23 nucleotides). This *B. malayi* miRNA subset (shown in blue) contains potential modulators of host gene expression. **(C) Sequence conservation between *B. malayi* ELV-origin miRNA seed sites and host ( *H. sapiens* ) miRNA seed sites**. miRNAs sharing perfectly conserved seed sites, defined here as nucleotides 2–8 of the mature miRNA, are marked (blue circles).

To more fully investigate the nucleic acid contents of these newly discovered vesicles, we carried out RNA-Seq with both L3 ELV and tissue-derived small RNAs. Reads generated by Illumina sequencing were processed and used to seed an miRNA discovery and abundance estimation pipeline using miRDeep2 [47] (read statistics and raw miRNA abundances can be found in S1 Table). To compare ELV and cellular RNA abundance, miRNA expression was normalized to the total miRNA read count within each sample. miRNA discovery and profiling was augmented with data from previously discovered miRNAs in closely related nematode species to help overcome gaps in the *B. malayi* draft genome assembly (see Methods). Fig 5A compares normalized miRNA expression between ELV and tissue for the 20 most abundant miRNAs in each sample. Although there is considerable conservation in relative miRNAs abundances, there are some notable observations and exceptions.

**Fig 6:**
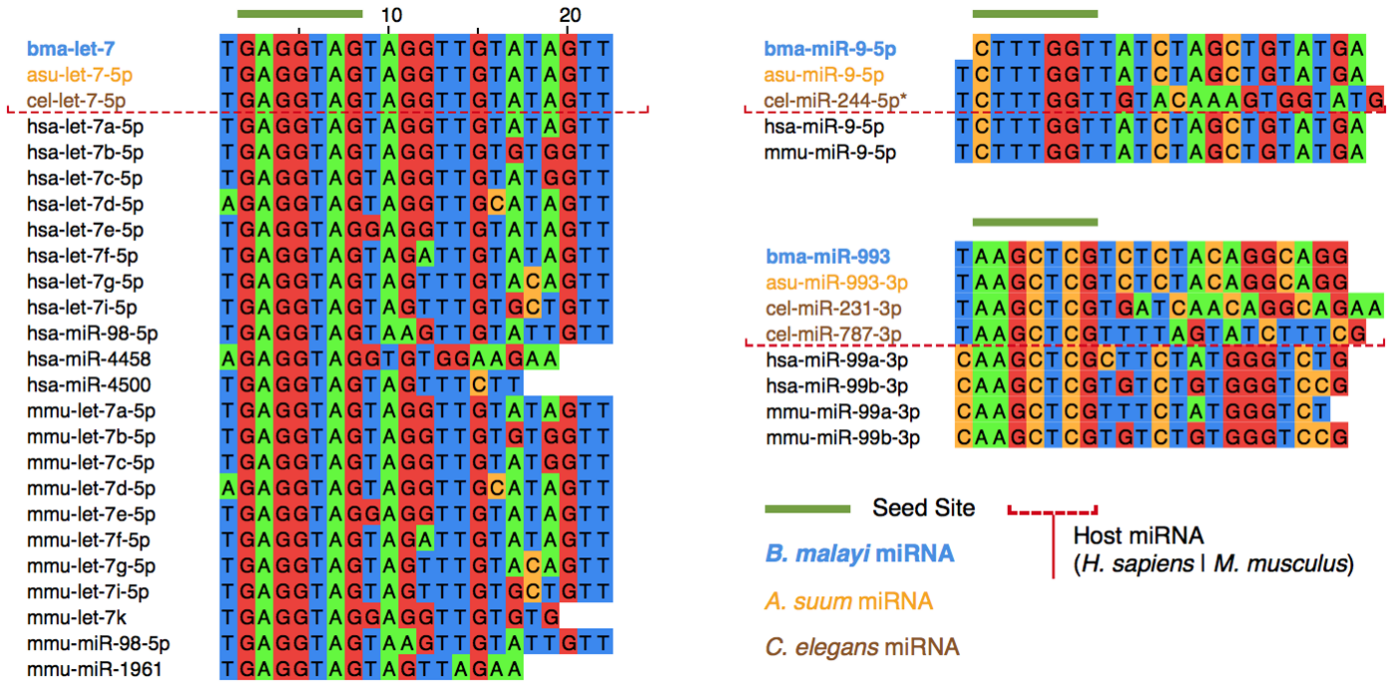
*Brugia malayi* ELV miRNA sequence homology to nematode and mammalian host miRNAs. miRNAs from *B. malayi*, *A. suum*, *C. elegans*, *H. sapiens*, and *M. musculus* were grouped by seed site sequence identity (nucleotides 2–8) for multiple sequence alignments. Alignments are shown for bma-let-7, bma-miR-9 and bma-miR-993. bma-let-7 is shown as an example of a *Brugia* ELV miRNA that exhibits both seed site and full length sequence conservation extending to other parasitic and free-living nematodes, as well as to mammalian host species. bma-miR-9 and bma-miR-993 are presented as examples where conserved parasite miRNAs have clear host homologs, yet lack one-to-one *C. elegans* orthologs. The complete set of alignments can be found in S1 Fig.

Bma-let-7 is significantly enriched in L3 ELVs as compared to L3 tissue, where it does not appear among the 20 most abundant miRNAs. Bma-let-7, along with four other *B. malayi* mature miRNAs found in ELVs (bma-miR-1, bma-miR-9, bma-miR-92, and bma-miR-100b), share perfect sequence identity with host (*Homo sapiens*) mature miRNAs, as shown in Fig 5B. Additionally, bma-miR-34 shares near perfect sequence identity with its *H. sapiens* homolog. 11 *B. malayi* miRNAs also share common seed sites with *H. sapiens* miRNAs (Fig 5C). *Brugia* ELV miRNA sequences were more broadly clustered by putative seed site and aligned to miRNAs from the soil-transmitted parasitic nematode *Ascaris suum*, the free living model nematode *Caenorhabditis elegans*, and mammalian host species *H. sapiens* and *Mus musculus* (Fig 6 and S1 Fig). In all cases, *Brugia* ELV miRNAs that share common seed sites with host miRNAs have one-to-one *A. suum* orthologs. In some cases, parasite miRNAs are better conserved in mammalian hosts than in *C. elegans* (e.g., bma-miR-9, bma-miR-993, and bma-miR-100b/c).

**Fig 7:**
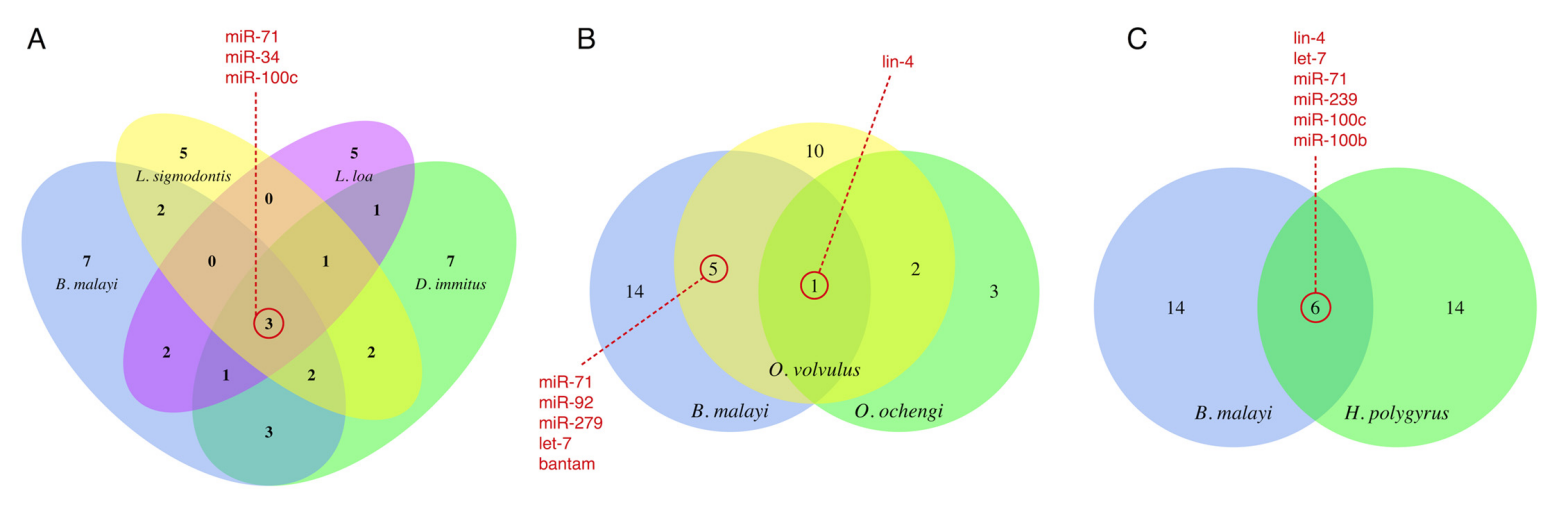
Comparison of the *B. malayi* ELV miRNA complement to miRNAs secreted by other parasitic nematodes species. (A & B) Comparison of the 20 most abundant *B. malayi* ELV miRNAs with the complements of miRNAs found circulating in the serum and plasma of definitive and model mammal hosts burdened with filarial infection (*Litomosoides sigmodontis* [26], *Dirofilaria immitis* [48], *Loa loa* [49], *Onchocerca volvulus* [48, 50], and *Onchocerca ochengi* [49]). The *D. immitis* miRNAs in (A) are restricted to the 20 most abundant miRNAs, and the *O. volvulus* miRNAs in (B) represent the combination of two non-overlapping sets arising from separate reports. (C) Comparison of the 20 most abundant miRNAs identified in *B. malayi* ELVs and *H. polygyrus* exosomes. These analyses reveal sets of common markers and a number of miRNAs unique to each species.

We examined the complement of the most abundant *Brugia* ELV-associated miRNAs with respect to very recent investigations of miRNAs released by other parasitic nematode species and found circulating in host biofluids [26, 48–50]. Common markers include let-7, lin-4, miR-34, miR-71, miR-92, and miR-100c (Fig 7A and 7B). While all members of this subset share seed site sequence identity with mammalian host miRNAs, lin-4, miR-34, miR-71, and miR-100c are sufficiently diverged from host miRNAs over their full length mature miRNA sequence and can potentially serve as biomarkers of filarial infection. Additionally, we compared the complements of the 20 most abundant *Brugia* ELV and *H. polygyrus* exosomal [26] miRNAs, identifying six miRNAs shared between these vesicles and a large number of miRNAs unique to each species (Fig 7C).

Enrichment of bma-let-7 and the high fractional presence of other parasite miRNAs sharing perfect or high homology to host miRNAs, leads us to speculate about a potential ELV-mediated mechanism by which parasite RNAs can be used to efficiently direct aspects of gene expression in host cells. Targets of endogenous let-7 family miRNAs in vertebrates include oncogenes, as well as genes involved in proliferation, apoptosis, and innate immunity [51–53]. Let-7 is intricately involved in macrophage polarization and responses to pathogen challenge [31, 33, 54], and the altering of host let-7 expression therefore represents a potentially advantageous point of intervention for an invading parasite. Live pathogens down-regulate the expression of let-7 family miRNAs, and let-7 miRNAs act on toll-like receptors (e.g. TLR4) that directly mediate macrophage responses [54–56]. Clearly, there is an important association between macrophage response to pathogens and let-7 expression. Our observation that *B. malayi* secrete let-7 and other potential modulators of host gene expression identifies a mechanism by which this host immune response might be manipulated. Supporting this hypothesis, let-7 and other miRNAs with host conservation have been identified in immunomodulatory *H. polygyrus* adult stage exosomes [26]. To fully dissect this hypothesis, a broad investigation of the interaction of ELV miRNAs and host immune cells *in vivo* is needed.

### *Brugia* ELVs are internalized by host macrophages

**Fig 8:**
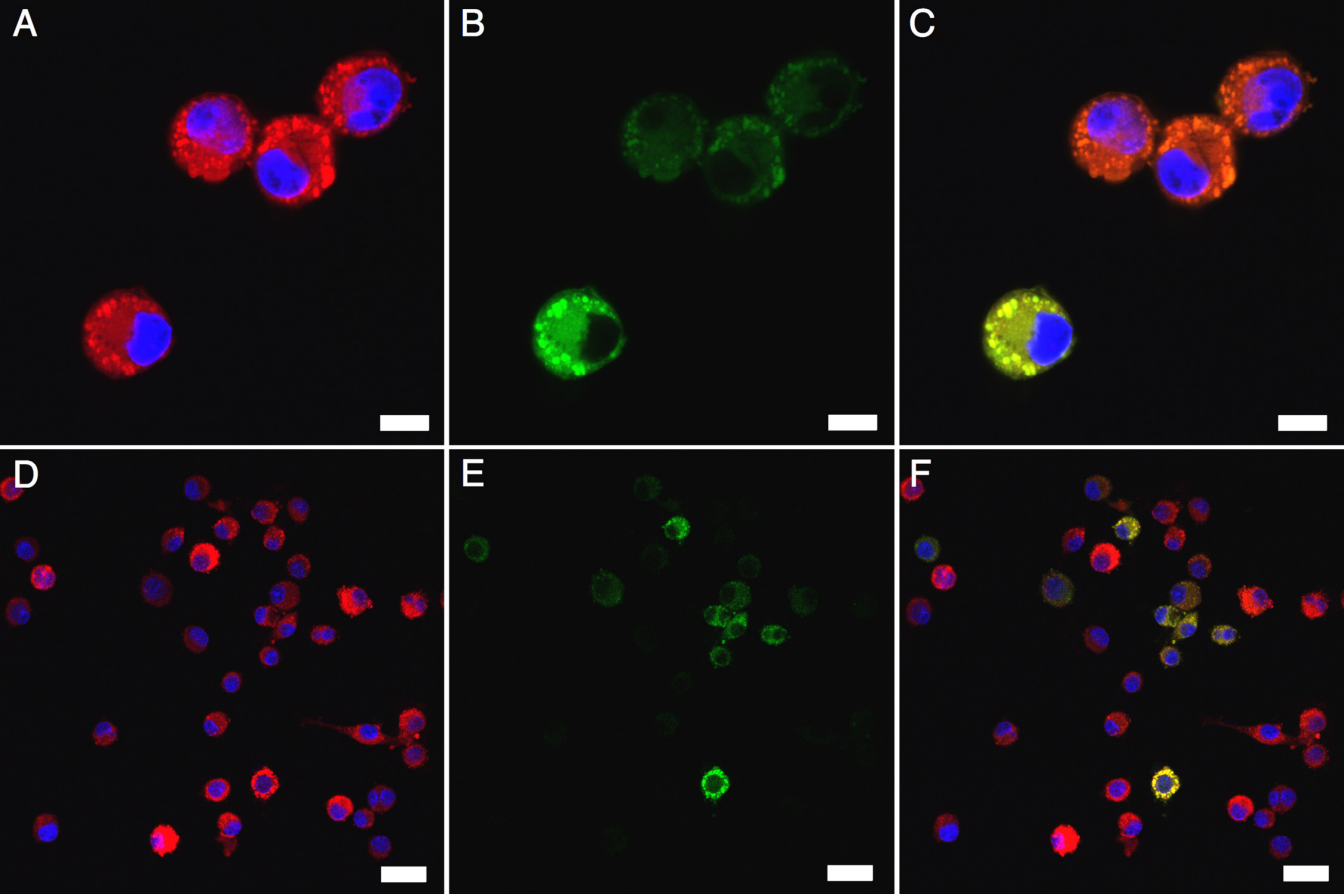
*Brugia* exosome-like vesicles (ELVs) are internalized by J774A.1 macrophages. (A and D) J774A.1 macrophages were labeled with PKH26 (red) and counterstained with DAPI (blue) to visualize nuclei. (B and E) *B. malayi* L3 stage ELVs were purified from a 24 hr parasite culture and labeled with PKH67 (green). 3 × 10^5^ J774A.1 were co-incubated with approximately 3 × 10^7^ labeled ELVs for 6 hrs at 37°C and washed repeatedly to remove unbound ELVs. Vesicles internalized by macrophages appear diffusely throughout cytoplasm and focused in discrete puncta associated with the cell membrane. (C and F) Merged images showing internalization of parasite ELVs. All images were acquired using a using a Leica TCS SP5 X Confocal/multiphoton microscope system with 20X (A-C) or 60X (D-F) objectives. Scale bars: 10 *μ*m (A-C) and 25 *μ*m (D-F).

Macrophages are critical mediators of the early immune response to invasive *Brugia* parasites [8]. To test the hypothesis that secreted *Brugia* ELVs interact with host macrophages, we used fluorescent lipophilic dyes to visualize the interaction between J774A.1 murine macrophages and ELVs. This cell line was chosen because it is commercially available, can be cultured readily and because it recapitulates the biology of primary macrophages and dendritic cells [57]. ELVs were labeled with PKH67, a green fluorescent dye, and incubated with J774A.1 labeled with PKH26, a red fluorescent dye. Confocal microscopy revealed efficient internalization of the ELVs by this macrophage cell line (Fig 8). Internalization was observed diffusely throughout the cell cytoplasm with focus around membrane-rich puncta associated with the surface of the macrophages (Fig 8B). This pattern of internalization is consistent with other studies describing a phagocytic route of vesicle internalization [58, 59]. Macrophages were counterstained with DAPI to determine the efficiency of cell labeling and ELV uptake. PKH26-labeling of J774A.1 was very efficient and all cells were visualized although intensity of labeling was variable (Fig 8D). Approximately 40–50% of macrophages internalized labeled ELVs to some degree (Fig 8E) with approximately 10% of macrophages internalizing ELVs at markedly higher levels (Fig 8E). There was no correlation between strong PKH 26-labelling of macrophages and vesicle uptake indicating internalization is not a factor of receptiveness to labeling.

### *Brugia* ELVs elicit a classically activated phenotype in host macrophages

**Fig 9:**
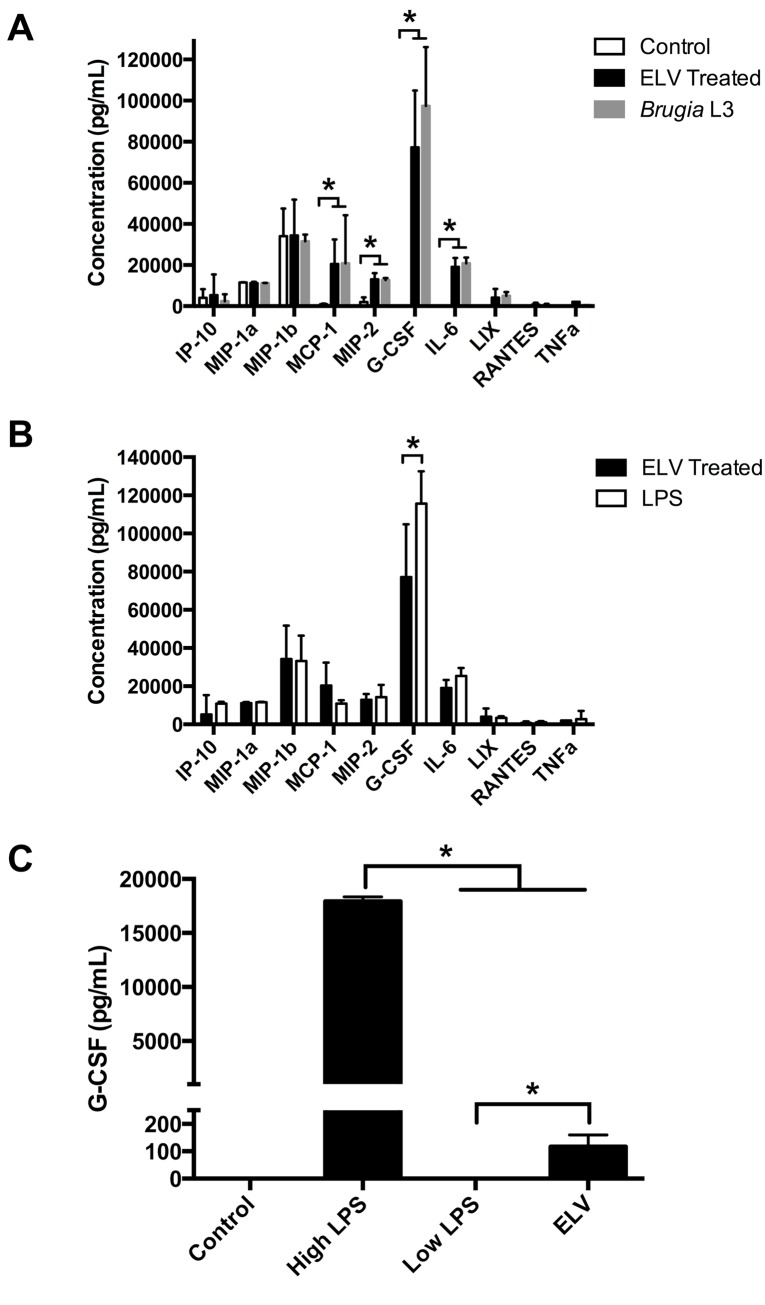
*Brugia* exosome-like vesicles (ELVs) elicit a classically activated phenotype in J774A.1 macrophages. (A) J774A.1 (5 × 10^5^) were treated with approximately 4 × 10^8^ purified L3 stage ELVs, live L3 stage parasites (10 worms) or naïve culture media (control) and supernatents collected after 48 hr. The presence of 32 cytokines/chemokines was simultaneously assayed using the Milliplex MAP Mouse Cytokine/Chemokine kit (EDM Millipore) interfaced with a Bio-Plex System (Bio-Rad) utilizing Luminex xMAP technology (Luminex). The quantification of identified cytokines is presented. The cytokine profile generated by ELV treatment is consistent with a classically activated phenotype. (B) Cytokine response to ELV treatment is compared to LPS (200 ng/mL). The close correlation of responses indicates ELV treatment generates a classically activated phenotype. (C) J774A.1 (5 × 10^5^) were treated with high dose LPS (200 ng/mL), low dose LPS (0.003 ng/mL), ELV or naïve culture media (control) for 24 hr, supernatant collected and assayed for G-CSF using a Mouse G-CSF Quantikine ELISA kit (R&D Systems). The absence of response to low dose LPS suggests the classically activated response is not due to LPS-like contamination.

Macrophage activation is dichotomous; classically activated macrophages (CAMΦ) are elicited by LPS or IFN-*γ* and have a generally pro-inflammatory phenotype whereas alternatively activated macrophages (AAMΦ), driven by IL-4 and IL-13, appear immunosuppressive or anti-inflammatory. Helminth infection is typically associated with the AAMΦ pathway although both CAMΦ and AAMΦ are involved in the immune response to, and immunopathology caused by, *Brugia* infection. Experiments demonstrate different *Brugia* preparations can generate both CAMΦ and AAMΦ activation phenotypes; dead and moribund worms and worm lysates produce CAMΦ [60] but live worms and complete excretory/secretory (ES) preparations drive AAMΦ [61–63]. To test the hypothesis that ELVs activate host macrophages, J774A.1 were treated with purified ELV preparations and their cytokine/chemokine responses monitored. J774A.1 were treated for 48 hrs with approximately 4 × 10^8^ L3 stage vesicles, purified from in vitro culture medium by ultracentrifugation. The macrophage response was assayed using the Milliplex MAP Mouse Cytokine/Chemokine kit (EDM Millipore) interfaced with a Bio-Plex System (Bio-Rad) utilizing Luminex xMAP technology, a platform capable of simultaneously identifying and quantifying 32 cytokines/chemokines. Vesicle treatment effectively activated J774A.1 macrophages with significant increases in G-CSF, MCP-1, IL-6 and MIP-2 levels compared to control macrophages treated with naïve RPMI 1640 culture media, (p ≤ 0.001)(Fig 9A). Smaller increases in LIX, RANTES and TNF-*α* were also noted. Healthy, viable L3 stage parasites produced an almost identical response (Fig 9A), the only difference being a modest but significant enhancement of G-CSF stimulation by the viable parasites (p < 0.001), suggesting that the dominant parasite immunogen(s) are found in the vesicle pellet. Finally, parasite culture media from which the ELVs had been removed by centrifugation did not produce this response, nor did live schistosomes (*S. mansoni* cercaria) or their secreted vesicles (S2 Fig) suggesting the *Brugia*-associated activation is specific to this parasite and not a general response to helminths or their secreted vesicles.

The activation profile observed would be considered more indicative of a CAMΦ response than AAMΦ; to confirm the response was CAMΦ-like, we compared it to the response elicited by LPS (200 ng/mL). The only significant differences were that ELV treatment stimulated G-CSF and IL-6 less effectively (p < 0.001) and stimulated MCP-1 more effectively (p < 0.001) than LPS (Fig 9B). The overall conservation of response, however, indicates these ELVs generate a CAMΦ phenotype. Since *Wolbachia*, the endosymbiont present in filarial nematodes, lack LPS biosynthetic capacity it seemed unlikely our CAMΦ-like response was driven by LPS-like contamination but to rule this out, endotoxin levels in our vesicle preparation were determined commercially (Lonza, Walkersville, MD). LPS-like activity was present (0.003 ng/mL) but at a concentration several orders of magnitude lower than the minimum dose required to stimulate J774A.1 macrophages [64]. As expected, treatment of macrophages with this low LPS dose was insufficient for activation (Fig 9C) indicating that the CAMΦ response we observe is not due to an LPS-like component in our preparation.

Since the stimulation of an AAMΦ phenotype by live *Brugia* and ES preparations thereof *in vivo* and *in vitro* has been well established [61–63] it might be expected that *Brugia* ELV preparations also stimulate a AAMΦ phenotype, especially since complete *Brugia* ES preparations are likely to include ELVs similar to those examined here, albeit at reduced concentrations. We observed a response consistent with a CAMΦ phenotype, however, although without the acute elevation in IL-*β* and TNF-*α* production others have seen in response to LPS [60]. One interpretation is that the CAMΦ > AAMΦ phenotype may be a somewhat artificial function of the homogenous J774A.1 monoculture used here as other studies describing a AAMΦ phenotype often use PBMC or other heterogeneous primary cell types. It would be instructive to monitor the responses of such mixed cell populations to the ELV preparation. Additionally, although the murine model is regarded as valuable for illuminating both how parasites establish themselves and the early host immune response, J774A.1 may not be optimal for studying this particular *Brugia*-host interaction and optimization with other murine or human cells may be required. Another interpretation, however, is that the purified ELVs examined here should be considered a distinct and specific fraction of the highly complex immunogenic facade presented by filarial parasites and may elicit a genuine CAMΦ phenotype when examined in isolation. Supporting this interpretation, exosomes isolated from other biological systems effectively generate a CAMΦ phenotype [59, 65, 66]. A key mediator of this pro-inflammatory response is Hsp70 [65], which was identified in our ELV proteomic profile. In summary, irrespective of the polarity of macrophage activation phenotype, our results unequivocally identify secreted ELVs as distinct parasite-derived structures capable of activating the host immune system.

A picture is emerging that parasitic helminths secrete functional exosome-like vesicles. The protein and small RNA cargo of these vesicles have putative effector functions at the host-parasite interface and potentially serve to create conditions favorable to the establishment or maintenance of infection. The identification of these cell-to-cell effector structures is exciting and prompts further investigation of their functional relevance. In particular, it will be important to describe the roles of individual miRNAs and proteins contained within the ELVs, to identify the host molecular targets being manipulated *in vivo*, and reveal any conserved or stage-specific effectors secreted across the parasite life cycle. Another intriguing question is whether or not there is any specificity or selectivity in host cells or tissues targeted and if so, what molecular mechanisms underscore this specificity. Addressing such questions will illuminate the fundamental interactions that occur between parasite and host, and may open previously unexploited opportunities for parasite control and diagnostics.

## Materials and Methods

### Mosquito maintenance

*Aedes aegypti* (Black eyed Liverpool strain, LVP), previously selected for susceptibility to infection with *Brugia malayi* [67], were maintained in controlled conditions (27°C ± 1°C and 75% ± 5% relative humidity) with a 16:8 photoperiod. Adult mosquitoes were fed a diet of 10% sucrose. Approximately 4,000 and 2,600 mosquitos were used for proteomics and RNA sequencing, respectively.

### Establishing *Brugia malayi* infection

For proteomics and transcriptomics, *B. malayi* microfilaria (mf) infected cat blood was obtained from the University of Georgia NIH/NIAID Filariasis Research Reagent Resource Center (FR3). Blood containing the parasites was diluted with defibrinated sheep’s blood (Hemostat Laboratories, CA, USA) to achieve a concentration of 80–100 mf per 20*μ*L. To establish infection, 3- to 5-day-old *Ae. Aegypti* (LVP) were allowed to feed for one hour on a glass membrane feeder. Mosquitoes were sucrose-starved for 24 hrs prior to blood feeding and those that did not take a blood meal were removed. Infected mosquitoes were maintained in the above described conditions for 13–15 days post infection (dpi) to allow development of parasites.

### *Brugia malayi* maintenance and collection of vesicle-containing media

In exploratory studies, larval (300 L3) and adult (30 male or 30 female) *B. malayi* were procured from the FR3. On arrival, parasites were cultured in 50 mL RPMI 1640 (Sigma-Aldrich, St. Louis, MO) at 37°C (5% CO_2_). Cell culture media was collected and replaced at 24 hr intervals for up to 72 hrs to collect secreted ELVs. For downstream sequencing and proteomics, *B. malayi* (13–15 dpi) were locally collected using methods described by FR3. Briefly, infected mosquitoes were immobilized by cooling to 4°C for 15 minutes. Immobilized mosquitoes were crushed in a mortar containing 5 ml of chilled Hanks’ balanced salt solution (HBSS, pH 7.0) containing pen-strep (0.4 units penicillin/ml, 0.4 mcg streptomycin/ml). Mosquitoes were then rinsed onto a 150 mesh sieve contained in a deep well plastic petri dish and washed 3–4 times using fresh chilled HBSS + pen-strep. Sieves were then placed into petri dishes containing warm (40°C) HBSS + pen-strep to allow infective larvae to migrate out. Sieves were transferred to new deep well petri dishes containing fresh warm HBSS every 30 minutes. Collected parasites were washed twice with warm HBSS + pen-strep, placed into 25 mL RPMI 1640 containing pen-strep (0.4 units penicillin/ml, 0.4 mcg streptomycin/ml) and held at 37°C, 5% CO_2_ for 24 hrs to collect secreted ELVs.

### Exosome-like vesicle purification

Differential centrifugation was used to isolate ELVs from 25 or 50 mL aliquots of *Brugia* culture media. Aliquots were collected from 24 hr incubations of larval or adult worms in culture media. Lower speed centrifugation and filtration steps were used to remove contaminating cells (300 × g, 10 mins) and cellular debris (10,000 × g, 15 mins). The resulting supernatants underwent filtration through 0.22 *μ*m filters and ultracentrifugation at 105,000 × g for 90 mins to pellet ELVs. Pellets were then washed with cold phosphate-buffered saline (PBS) and a final spin was carried out at 105,000 × g for 90 mins. Supernatants were discarded and pellets were resuspended in small volumes (30–250 uL) of PBS for imaging, sequencing, and proteomics, and RPMI for immunological assays. Samples were kept on ice and centrifugation steps were carried out at 4°C. Resuspended ELVs were stored at −80°C.

### Electron microscopy and nanoparticle tracking analysis

Small aliquots of ELV suspension (3 *μ*l) were applied to carbon coated 200 mesh copper grids and negatively stained with 2% uranyl acetate. Images were taken using a JEOL 2100 scanning and transmission electron microscope (Japan Electron Optics Laboratories, Akishima, Japan) at the Microscopy and NanoImaging Facility (Iowa State University). Nanoparticle tracking analysis was carried out with the NanoSight LM10 (NanoSight Ltd., Amesbury, UK) to ascertain the size and frequency distribution of individual vesicle preparations, assayed in triplicate. The Brownian motion of particles in solution is related back to particle sizes and numbers, allowing better statistical resolution of vesicle size and concentration [68].

### LC-MS/MS and proteomic analysis

Protein was isolated from purified exosome-like vesicles for proteomic analysis (System Biosciences). Briefly, samples were modified with 10% SDS to a final concentration of 2% SDS, heated at 100°C for 15 minutes and clarified by centrifugation. Protein concentration was determined using a Qubit fluorometry assay (Invitrogen). 15 *μ*g of material was processed by SDS-PAGE using a 10% Bis-Tris homogeneous gel and the MES buffer system. In-gel digestion with trypsin was done at 37°C for 4 hrs using a ProGest robot (DigiLab, Marlborough, MA). The digested sample was analyzed by nano LC-MS/MS analysis using a Waters NanoAcquity HPLC system interfaced to a ThermoFisher Q Exactive. Data were searched against a copy of the *B. malayi* UniProt database (taxon ID: 6278) using a locally running copy of MASCOT (Matrix Science Ltd., London, UK). The search was restricted using the following parameters; maximum missed cleavages = 2, fixed modifications = carbamidomethyl (C), variable modifications = Oxidation (M), Acetyl (N-term), Pyro-Glu (N-term Q) and Deamidation (N, Q), a peptide mass tolerance of 10 ppm, and a fragment mass tolerance of 0.02 Da. Mascot DAT files were parsed into the Scaffold software for validation, filtering and to create a nonredundant list per sample. Data were filtered using a minimum protein value of 90%, a minimum peptide value of 50% (Prophet scores) and requiring at least two unique peptides per protein.

### RNA isolation and sequencing

For detection of RNA species in ELV preparations, small RNAs were preferentially isolated from vesicle-containing pellets using the miRCURY RNA Isolation Kit (Exiqon, Vedbaek, Denmark) and RNA samples were examined with an Agilent 2100 Bioanalyzer using the RNA 6000 Nano Kit. For small RNA sequencing (RNA-Seq), total RNA was isolated from ELVs released by ∼5,000 L3s over a 24 hr incubation period using the Total RNA and Protein Isolation Kit (Invitrogen, Carlsbad, CA). In parallel, total RNA was isolated from whole worm tissue using a TRIzol (Invitrogen) protocol, where a 6 hr precipitation step was carried out at -80°C to improve small RNA recovery. RNA NGS libraries were constructed using modified Illumina adapter methods using SBI’s XRNA Sample Preparation Kit (System Biosciences, Mountain View, CA) and indexed with separate bar codes for multiplex sequencing on an Illumina MiSeq v3 instrument using a 2 × 75 bp paired end run setting.

### miRNA discovery and abundance estimation

Raw reads were trimmed to remove adapter sequences, filtered by quality score, and de-multiplexed using the FASTX-Toolkit [69] (sequencing data are deposited with the NCBI SRA under project number PRJNA285132). The miRDeep2 pipeline was used to map short RNA reads (>15 nt) to the *B. malayi* genome for miRNA discovery, and to estimate and normalize miRNA abundances with respect to total miRNA read count. Nematode precursor and mature miRNA sequences deposited into miRBase [70] were used in the pipeline, including known *B. pahangi*, *Caenorhabditis elegans*, *Ascaris suum*, *Haemonchus contortus*, and *Strongyloides ratti* miRNAs. Non-mapped reads were ranked by abundance, filtered for homology against known miRNAs in the phylum Nematoda using BLASTn [71], and incorporated for final quantification of abundance with the miRDeep quantifier script, allowing for capture of miRNAs that did not map to the *B. malayi* assembly due to sequencing gaps. The ggplot2 package [72] of the statistical programming language *R* was used to organize and visualize comparisons between vesicular and tissue RNA samples.

### Cell culture

J774A.1 murine macrophages (ATCC, Manassas, VA) were maintained in complete tissue culture medium (Dulbecco’s modified Eagle’s medium, 25 mM HEPES, pH 7.4 supplemented with 2 mM L-glutamine, 100 U/mL penicillin, 100 *μ*g/mL streptomycin, 0.05 *μ*M 2-mercaptoethanol, and 10% heat-inactivated fetal bovine serum) at 37°C and 5% CO_2_. 24 hrs prior to assays, 400 *μ*L cells were plated in standard 24-well plates at a density of 5 × 10^5^ cells/well.

### Vesicle labeling and uptake

Exosome-like vesicles were purified from a 24 hr culture of 300 *Brugia malayi* L3 parasites as described above and labeled with the green fluorescent dye, PKH67 (Sigma-Aldrich, St Louis, MO,USA), according to the manufacturer’s instructions. ELVs were incubated with PKH67 for 5 min at room temperature and the reaction terminated by addition of 1% BSA in PBS. RPMI 1640 media was added, mixed and centrifuged at 105,000 × g for 1 hr to separate ELV-bound PKH67 from excess PKH67. Labeled ELV were washed again then resuspended in an appropriate volume of complete tissue culture medium (Dulbecco’s modified Eagles medium, 25 mM HEPES, pH 7.4 supplemented with 2 mM L-glutamine, 100 U/mL penicillin, 100 *μ*g/mL streptomycin, 0.05 *μ*M 2-mercaptoethanol and 10% heat-inactivated fetal bovine serum).

J774A.1 were labeled with red fluorescent lipophilic dye, PKH26 (Sigma-Aldrich, St Louis, MO), according to the manufacturer’s instructions. Macrophages were incubated with PKH26 for 5 min at room temperature and the reaction terminated by addition of 1% BSA. To remove excess unbound dye, samples were centrifuged at 400 × g for 10 minutes at room temperature and the supernatant discarded. Centrifugation was repeated three more times using 10 ml of complete media to ensure full removal of unbound dye and the cells were re-suspended in 1 mL of complete medium. Approximately 3 × 10^5^ labeled cells were plated onto sterile coverslips and incubated overnight at 37°C/5% CO_2_. Labeled ELV suspension (approximately 3 × 10^7^ per coverslip) was added to labeled J774A.1 and incubated for 6 hrs. Cells were washed 5 times with ice-cold PBS to remove excess labeled ELVs, the cells fixed in 4% paraformaldehyde (Sigma-Aldrich), washed and counterstained with DAPI before mounting and storage at 4°C. Preparations were visualized using a Leica TCS SP5 X Confocal/multiphoton microscope system (Leica Microsystems Inc., Buffalo Grove, IL).

### Detection of macrophage modulation by Luminex assay

Triplicate wells of adhered J774A.1 were treated with approximately 4 × 10^8^ purified L3 stage ELVs. The ELVs were purified by ultracentrifugation as previously described, resuspended in RPMI 1640 medium (Gibco/Life Technologies, Carlsbad, CA) and quantified by nanoparticle tracking analysis. Other treatments were similar volumes of vesicle depleted L3 culture medium (supernatant created following pelleting of ELV fraction from spent parasite culture medium), live *B. malayi* L3 parasites (10 worms/well), lipopolysaccharide (LPS; final concentration 200 ng/mL)(Sigma-Aldrich, St. Louis, MO), naïve RPMI 1640 culture medium and various combinations of these conditions. Supernatants from these cell cultures (400 *μ*L/well) were collected 24 or 48 hrs post-treatment and centrifuged briefly (2,000 × g for 10 min) to remove non-adhered cells and cell debris before being analyzed for the presence of cytokines/chemokines. The Milliplex MAP Mouse Cytokine/Chemokine kit (EDM Millipore, Billerica, MA) interfaced with a Bio-Plex System (Bio-Rad, Hercules, CA) utilizing Luminex xMAP technology (Luminex, Austin, TX) allowed the simultaneous identification and quantification of the following analytes in the cell culture supernatant: Eotaxin, G-CSF, GM-CSF, IFN*γ*, IL-1*α*, M-CSF, IL-1*β*, IL-2, IL-3, IL-4, IL-5, IL-6, IL-7, IL-10, IL-12(p40), IL-13, IL-15, IL-17, IP-10, MIP-2, KC, LIF, LIX, MCP-1, MIP-1*α*, MIP-1*β*, MIG, RANTES, TNF*α*, IL-12(p70), VEGF, IL-9. Briefly, experimental samples, background, standards and controls were added to a 96-well plate and combined with equal volumes of pre-mixed, antibody coated magnetic beads; the plate was sealed and incubated overnight at 4°C. Following washing, 25 *μ*L of detection antibody was added and the plate incubated for one hour at room temperature with shaking. Streptavidin-Phycoerythrin (25 *μ*L) was added to each well and the plate incubated for a further hour at room temperature before washing. Finally, 150 *μ*L assay buffer was added to all wells and fluorescence immediately recorded. Median fluorescent intensity data were analyzed as recommended using a five-parameter logistic curve-fitting method for calculating cytokine/chemokine concentration.

### G-CSF ELISA

Triplicate wells of adhered J774A.1 cells, prepared as described above, were treated with LPS (final concentration 200 ng/mL or 0.003 ng/mL), approximately 4 × 10^8^ purified L3 stage ELVs as described above, or RPMI 1640 as negative control. Cell culture supernatants were collected 24 hrs after treatment, cleared via centrifugation as described previously and assayed for G-CSF using a Mouse G-CSF Quantikine ELISA kit (R&D Systems, Minneapolis, MN). Standard curves were generated using Prism 6 software (GraphPad Software, San Diego, CA) and sample G-CSF concentrations determined by regression analysis.

### Statistical analysis

For analysis of Luminex data, Tukey’s test was used to compare overall treatments while multiple t-tests, incorporating the Holm-Sidak method to correct for multiple comparisons, were used to compare individual chemokines/cytokines following treatments. t-tests were used to compare treatment groups following ELISA analysis. All statistical analyses were performed using Prism 6 for Mac (Graphpad).

## Supporting Information

S1 Video *Brugia malayi* L3 ELVs recorded via NanoSight. (MP4)

S1 Table Small RNA-Seq miRNA abundances and read statistics. (XLSX)

S1 Fig *Brugia malayi* ELV miRNA sequence homology to nematode and mammalian host miRNAs. miRNAs are grouped by putative seed site and aligned.
(TIFF)

S2 Fig Macrophage activation is a specific function of *Brugia* ELVs. (A) J774A.1 macrophages (5 × 10^5^) were treated with approximately 4 × 10^8^ purified L3 stage ELVs, an equivalent volume of culture media supernatant from which ELVs had been depleted by centrifugation (SN Treated) or naïve culture media (control). The presence of 32 cytokines/chemokines was simultaneously assayed using the Milliplex MAP Mouse Cytokine/Chemokine kit (EDM Millipore) interfaced with a Bio-Plex System (Bio-Rad) utilizing Luminex xMAP technology (Luminex). The quantification of identified cytokines is presented. ELV treatment, but not the ELV depleted culture media, generates a classically activated phenotype. (B) J774A.1 macrophages were treated with approximately 4 × 10^8^ ELVs collected from a culture of *Schistosoma mansoni* invasive stage schistosomules as described for *Brugia* (*Sm* ELV), live *S. mansoni* schistosomules (300 per well; *Sm*) and naïve RPMI 1640 culture media (control). Macrophages were not activated by either schistosome preparation.
(TIFF)

## References

[1] Global programme to eliminate lymphatic filariasis: progress report, 2011. Weekly Epidemiological Record. 2012 9;87(37):346–356. 22977953

[2] Bartholomay LC, Christensen BM. Vector-parasite interactions in mosquito-borne filariasis Klei TR, Rajan TV, editors. In: The Filaria. Springer Science & Business Media; 2002.

[3] Erickson SM, Xi Z, Mayhew GF, Ramirez JL, Aliota MT, Christensen BM, et al Mosquito infection responses to developing filarial worms. PLoS Neglected Tropical Diseases. 2009;3(10):e529 10.1371/journal.pntd.0000529 19823571 PMC2752998

[4] Allen JE, Maizels RM. Diversity and dialogue in immunity to helminths. Nature reviews Immunology. 2011 6;11(6):375–388. 10.1038/nri2992 21610741

[5] Maizels RM, Pearce EJ, Artis D, Yazdanbakhsh M, Wynn TA. Regulation of pathogenesis and immunity in helminth infections. The Journal of Experimental Medicine. 2009 9;206(10):2059–2066. 10.1084/jem.20091903 19770272 PMC2757871

[6] van Riet E, Hartgers FC, Yazdanbakhsh M. Chronic helminth infections induce immunomodulation: Consequences and mechanisms. Immunobiology. 2007 6;212(6):475–490. 10.1016/j.imbio.2007.03.009 17544832

[7] Hoerauf A, Satoguina J, Saeftel M, Specht S. Immunomodulation by filarial nematodes. Parasite Immunology. 2005 10;27(10–11):417–429. 10.1111/j.1365-3024.2005.00792.x 16179035

[8] Devaney E, Osborne J. The third-stage larva (L3) of *Brugia*: its role in immune modulation and protective immunity. Microbes and Infection. 2000 9;2(11):1363–1371. 10.1016/S1286-4579(00)01290-9 11018453

[9] Zang X, Yazdanbakhsh M, Jiang H, Kanost MR, Maizels RM. A novel serpin expressed by blood-borne microfilariae of the parasitic nematode *Brugia malayi* inhibits human neutrophil serine proteinases. Blood. 1999 8;94(4):1418–1428. 10438730

[10] Zang X, Atmadja AK, Gray P, Allen JE, Gray CA, Lawrence RA, et al The serpin secreted by *Brugia malayi* microfilariae, Bm-SPN-2, elicits strong, but short-lived, immune responses in mice and humans. Journal of immunology (Baltimore, Md: 1950). 2000 11;165(9):5161–5169. 10.4049/jimmunol.165.9.5161 11046048

[11] Falcone FH, Loke P, Zang X, MacDonald AS, Maizels RM, Allen JE. A *Brugia malayi* homolog of macrophage migration inhibitory factor reveals an important link between macrophages and eosinophil recruitment during nematode infection. Journal of immunology (Baltimore, Md: 1950). 2001 11;167(9):5348–5354. 10.4049/jimmunol.167.9.5348 11673551

[12] Zang X, Taylor P, Wang JM, Meyer DJ, Scott AL, Walkinshaw MD, et al Homologues of human macrophage migration inhibitory factor from a parasitic nematode. Gene cloning, protein activity, and crystal structure. The Journal of biological chemistry. 2002 11;277(46):44261–44267. 10.1074/jbc.M204655200 12221083

[13] Hewitson JP, Harcus YM, Curwen RS, Dowle AA, Atmadja AK, Ashton PD, et al The secretome of the filarial parasite, *Brugia malayi*: proteomic profile of adult excretory-secretory products. Molecular and Biochemical Parasitology. 2008 7;160(1):8–21. 10.1016/j.molbiopara.2008.02.007 18439691

[14] Moreno Y, Geary TG. Stage- and gender-specific proteomic analysis of *Brugia malayi* excretory-secretory products. PLoS Neglected Tropical Diseases. 2008;2(10):e326 10.1371/journal.pntd.0000326 18958170 PMC2569413

[15] Bennuru S, Semnani R, Meng Z, Ribeiro JMC, Veenstra TD, Nutman TB. *Brugia malayi* excreted/secreted proteins at the host/parasite interface: stage- and gender-specific proteomic profiling. PLoS Neglected Tropical Diseases. 2009;3(4):e410 10.1371/journal.pntd.0000410 19352421 PMC2659452

[16] Geary J, Satti M, Moreno Y, Madrill N, Whitten D, Headley SA, et al First analysis of the secretome of the canine heartworm, *Dirofilaria immitis* . Parasites & vectors. 2012;5(1):140 10.1186/1756-3305-5-140 22781075 PMC3439246

[17] Tawill S, Le Goff L, Ali F, Blaxter M, Allen JE. Both free-living and parasitic nematodes induce a characteristic Th2 response that is dependent on the presence of intact glycans. Infection and immunity. 2004 1;72(1):398–407. 10.1128/IAI.72.1.398-407.2004 14688121 PMC343992

[18] Valadi H, Ekström K, Bossios A, Sjöstrand M, Lee JJ, Lötvall JO. Exosome-mediated transfer of mRNAs and microRNAs is a novel mechanism of genetic exchange between cells. Nature cell biology. 2007 6;9(6):654–659. 10.1038/ncb1596 17486113

[19] Mittelbrunn M, Gutiérrez-Vázquez C, Villarroya-Beltri C, González S, Sánchez-Cabo F, González MÁ, et al Unidirectional transfer of microRNA-loaded exosomes from T cells to antigen-presenting cells. Nature communications. 2011;2:282 10.1038/ncomms1285 21505438 PMC3104548

[20] Vickers KC, Palmisano BT, Shoucri BM, Shamburek RD, Remaley AT. Corrigendum: MicroRNAs are transported in plasma and delivered to recipient cells by high-density lipoproteins. Nature cell biology. 2014 12;17(1):104–104. 10.1038/ncb3074 PMC3074610 21423178

[21] Montecalvo A, Larregina AT, Shufesky WJ, Stolz DB, Sullivan MLG, Karlsson JM, et al Mechanism of transfer of functional microRNAs between mouse dendritic cells via exosomes. Blood. 2012 1;119(3):756–766. 10.1182/blood-2011-02-338004 22031862 PMC3265200

[22] Chen X, Liang H, Zhang J, Zen K, Zhang CY. Secreted microRNAs: a new form of intercellular communication. Trends in cell biology. 2012 3;22(3):125–132. 10.1016/j.tcb.2011.12.001 22260888

[23] Kalra H, Simpson RJ, Ji H, Aikawa E, Altevogt P, Askenase P, et al Vesiclepedia: a compendium for extracellular vesicles with continuous community annotation. PLoS biology. 2012;10(12):e1001450 10.1371/journal.pbio.1001450 23271954 PMC3525526

[24] van Niel G, Porto-Carreiro I, Simoes S, Raposo G. Exosomes: a common pathway for a specialized function. Journal of biochemistry. 2006 7;140(1):13–21. 10.1093/jb/mvj128 16877764

[25] Lakkaraju A, Rodriguez-Boulan E. Itinerant exosomes: emerging roles in cell and tissue polarity. Trends in cell biology. 2008 5;18(5):199–209. 10.1016/j.tcb.2008.03.002 18396047 PMC3754907

[26] Buck AH, Coakley G, Simbari F, McSorley HJ, Quintana JF, Le Bihan T, et al Exosomes secreted by nematode parasites transfer small RNAs to mammalian cells and modulate innate immunity. Nature communications. 2014;5:5488 10.1038/ncomms6488 25421927 PMC4263141

[27] Twu O, de Miguel N, Lustig G, Stevens GC, Vashisht AA, Wohlschlegel JA, et al *Trichomonas vaginalis* Exosomes Deliver Cargo to Host Cells and Mediate Host Parasite Interactions. PLoS Pathogens. 2013;9(7):e1003482 10.1371/journal.ppat.1003482 23853596 PMC3708881

[28] Marcilla A, Trelis M, Cortés A, Sotillo J, Cantalapiedra F, Minguez MT, et al Extracellular vesicles from parasitic helminths contain specific excretory/secretory proteins and are internalized in intestinal host cells. PLoS ONE. 2012;7(9):e45974 10.1371/journal.pone.0045974 23029346 PMC3454434

[29] Bernal D, Trelis M, Montaner S, Cantalapiedra F, Galiano A, Hackenberg M, et al Surface analysis of *Dicrocoelium dendriticum*. The molecular characterization of exosomes reveals the presence of miRNAs. Journal of proteomics. 2014 6;105:232–241. 10.1016/j.jprot.2014.02.012 24561797

[30] Silverman JM, Clos J, de’Oliveira CC, Shirvani O, Fang Y, Wang C, et al An exosome-based secretion pathway is responsible for protein export from *Leishmania* and communication with macrophages. Journal of cell science. 2010 3;123(Pt 6):842–852. 10.1242/jcs.056465 20159964

[31] Chen XM, Splinter PL, O’Hara SP, LaRusso NF. A cellular micro-RNA, let-7i, regulates Toll-like receptor 4 expression and contributes to cholangiocyte immune responses against *Cryptosporidium parvum* infection. The Journal of biological chemistry. 2007 9;282(39):28929–28938. 10.1074/jbc.M702633200 17660297 PMC2194650

[32] Kumar M, Ahmad T, Sharma A, Mabalirajan U, Kulshreshtha A, Agrawal A, et al Let-7 microRNA-mediated regulation of IL-13 and allergic airway inflammation. The Journal of allergy and clinical immunology. 2011 11;128(5):1077–85. e1–10 10.1016/j.jaci.2011.04.034 21616524

[33] Banerjee S, Xie N, Cui H, Tan Z, Yang S, Icyuz M, et al MicroRNA let-7c regulates macrophage polarization. Journal of immunology (Baltimore, Md: 1950). 2013 6;190(12):6542–6549. 10.4049/jimmunol.1202496 PMC3679284 23667114

[34] Lodish HF, Zhou B, Liu G, Chen CZ. Micromanagement of the immune system by microRNAs. Nature reviews Immunology. 2008 2;8(2):120–130. 10.1038/nri2252 18204468

[35] Simpson RJ, Kalra H, Mathivanan S. ExoCarta as a resource for exosomal research. Journal of extracellular vesicles. 2012;1(0):569 10.3402/jev.v1i0.18374 PMC3760644 24009883

[36] Chen CY, Hogan MC, Ward CJ. Purification of exosome-like vesicles from urine. Methods in enzymology. 2013;524:225–241. 10.1016/B978-0-12-397945-2.00013-5 23498743 PMC4028690

[37] Bennuru S, Semnani R, Meng Z, Ribeiro JM, Veenstra TD, Nutman TB. *Brugia malayi* excreted/secreted proteins at the host/parasite interface: stage-and gender-specific proteomic profiling. PLoS neglected tropical diseases. 2009;3(4):e410 10.1371/journal.pntd.0000410 19352421 PMC2659452

[38] Huntley RP, Sawford T, Mutowo-Meullenet P, Shypitsyna A, Bonilla C, Martin MJ, et al The GOA database: Gene Ontology annotation updates for 2015. Nucleic Acids Research. 2015 1;43(Database issue):D1057–63. 10.1093/nar/gku1113 25378336 PMC4383930

[39] Binns D, Dimmer E, Huntley R, Barrell D, O’Donovan C, Apweiler R. QuickGO: a web-based tool for Gene Ontology searching. Bioinformatics. 2009 11;25(22):3045–3046. 10.1093/bioinformatics/btp536 19744993 PMC2773257

[40] Guiliano DB, Hong X, McKerrow JH, Blaxter ML, Oksov Y, Liu J, et al A gene family of cathepsin L-like proteases of filarial nematodes are associated with larval molting and cuticle and eggshell remodeling. Molecular and biochemical parasitology. 2004;136(2):227–242. 10.1016/j.molbiopara.2004.03.015 15478801

[41] Ford L, Zhang J, Liu J, Hashmi S, Fuhrman JA, Oksov Y, et al Functional analysis of the cathepsin-like cysteine protease genes in adult *Brugia malayi* using RNA interference. PLoS Neglected tropical diseases. 2009;3(2):e377 10.1371/journal.pntd.0000377 19190745 PMC2634747

[42] Song C, Gallup JM, Day TA, Bartholomay LC, Kimber MJ. Development of an in vivo RNAi protocol to investigate gene function in the filarial nematode, *Brugia malayi* . PLoS pathogens. 2010;6(12):e1001239 10.1371/journal.ppat.1001239 21203489 PMC3009605

[43] Lustigman S, Zhang J, Liu J, Oksov Y, Hashmi S. RNA interference targeting cathepsin L and Z-like cysteine proteases of *Onchocerca volvulus* confirmed their essential function during L3 molting. Molecular and biochemical parasitology. 2004;138(2):165–170. 10.1016/j.molbiopara.2004.08.003 15555728

[44] Rhoads M, Fetterer R. Developmentally regulated secretion of cathepsin L-like cysteine proteases by *Haemonchus contortus* . The Journal of parasitology. 1995;p. 505–512. 10.2307/3283844 7623189

[45] Dalton JP, Neill SO, Stack C, Collins P, Walshe A, Sekiya M, et al *Fasciola hepatica* cathepsin L-like proteases: biology, function, and potential in the development of first generation liver fluke vaccines. International journal for parasitology. 2003;33(11):1173–1181. 10.1016/S0020-7519(03)00171-1 13678633

[46] Marcilla A, Trelis M, Cortés A, Sotillo J, Cantalapiedra F, Minguez MT, et al Extracellular vesicles from parasitic helminths contain specific excretory/secretory proteins and are internalized in intestinal host cells. PLoS One. 2012;7(9):e45974 10.1371/journal.pone.0045974 23029346 PMC3454434

[47] Friedländer MR, Mackowiak SD, Li N, Chen W, Rajewsky N. miRDeep2 accurately identifies known and hundreds of novel microRNA genes in seven animal clades. Nucleic Acids Research. 2012 1;40(1):37–52. 10.1093/nar/gkr688 21911355 PMC3245920

[48] Tritten L, Burkman E, Moorhead A, Satti M, Geary J, Mackenzie C, et al Detection of circulating parasite-derived microRNAs in filarial infections. PLoS neglected tropical diseases. 2014;8(7):e2971 10.1371/journal.pntd.0002971 25033073 PMC4102413

[49] Tritten L, O’Neill M, Nutting C, Wanji S, Njouendoui A, Fombad F, et al *Loa loa* and *Onchocerca ochengi* miRNAs detected in host circulation. Molecular and biochemical parasitology. 2014;198(1):14–17. 10.1016/j.molbiopara.2014.11.001 25461483

[50] Quintana JF, Makepeace BL, Babayan SA, Ivens A, Pfarr KM, Blaxter M, et al Extracellular *Onchocerca*-derived small RNAs in host nodules and blood. Parasites & vectors. 2015;8(1):58 10.1186/s13071-015-0656-1 25623184 PMC4316651

[51] Johnson SM, Grosshans H, Shingara J, Byrom M, Jarvis R, Cheng A, et al RAS Is Regulated by the let-7 MicroRNA Family. Cell. 2005 3;120(5):635–647. 10.1016/j.cell.2005.01.014 15766527

[52] Johnson CD, Esquela-Kerscher A, Stefani G, Byrom M, Kelnar K, Ovcharenko D, et al The let-7 microRNA represses cell proliferation pathways in human cells. Cancer research. 2007 8;67(16):7713–7722. 10.1158/0008-5472.CAN-07-1083 17699775

[53] Roush S, Slack FJ. The let-7 family of microRNAs. Trends in cell biology. 2008 10;18(10):505–516. 10.1016/j.tcb.2008.07.007 18774294

[54] Schulte LN, Eulalio A, Mollenkopf HJ, Reinhardt R, Vogel J. Analysis of the host microRNA response to *Salmonella* uncovers the control of major cytokines by the let-7 family. The EMBO Journal. 2011 5;30(10):1977–1989. 10.1038/emboj.2011.94 21468030 PMC3098495

[55] Androulidaki A, Iliopoulos D, Arranz A, Doxaki C, Schworer S, Zacharioudaki V, et al The Kinase Akt1 Controls Macrophage Response to Lipopolysaccharide by Regulating MicroRNAs. Immunity. 2009 8;31(2):220–231. 10.1016/j.immuni.2009.06.024 19699171 PMC2865583

[56] Liu G, Abraham E. MicroRNAs in immune response and macrophage polarization. Arteriosclerosis, Thrombosis, and Vascular Biology. 2013 2;33(2):170–177. 10.1161/ATVBAHA.112.300068 23325473 PMC3549532

[57] van Helden SFG, van Leeuwen FN, Figdor CG. Human and murine model cell lines for dendritic cell biology evaluated. Immunology letters. 2008 5;117(2):191–197. 10.1016/j.imlet.2008.02.003 18384885

[58] Feng D, Zhao WL, Ye YY, Bai XC, Liu RQ, Chang LF, et al Cellular internalization of exosomes occurs through phagocytosis. Traffic. 2010;11(5):675–687. 10.1111/j.1600-0854.2010.01041.x 20136776

[59] Atay S, Gercel-Taylor C, Taylor DD. Human Trophoblast-Derived Exosomal Fibronectin Induces Pro-Inflammatory Il-1*β* Production by Macrophages. American Journal of Reproductive Immunology. 2011;66(4):259–269. 10.1111/j.1600-0897.2011.00995.x 21410811

[60] Taylor MJ, Cross HF, Bilo K. Inflammatory responses induced by the filarial nematode *Brugia malayi* are mediated by lipopolysaccharide-like activity from endosymbiotic *Wolbachia* bacteria. The Journal of Experimental Medicine. 2000 4;191(8):1429–1436. 10.1084/jem.191.8.1429 10770808 PMC2193140

[61] Osborne J, Hunter SJ, Devaney E. Anti-interleukin-4 modulation of the Th2 polarized response to the parasitic nematode *Brugia pahangi* . Infection and immunity. 1996;64(9):3461–3466. 8751885 10.1128/iai.64.9.3461-3466.1996 PMC174249

[62] Allen JE, Macdonald AS. Profound suppression of cellular proliferation mediated by the secretions of nematodes. Parasite immunology. 1998;20(5):241–247. 10.1046/j.1365-3024.1998.00151.x 9651925

[63] Weinkopff T, Mackenzie C, Eversole R, Lammie PJ. Filarial Excretory-Secretory Products Induce Human Monocytes to Produce Lymphangiogenic Mediators. PLoS neglected tropical diseases. 2014;8(7):e2893 10.1371/journal.pntd.0002893 25010672 PMC4091784

[64] Amano F, Akamatsu Y. A lipopolysaccharide (LPS)-resistant mutant isolated from a macrophagelike cell line, J774.1, exhibits an altered activated-macrophage phenotype in response to LPS. Infection and immunity. 1991 6;59(6):2166–2174. 1645329 10.1128/iai.59.6.2166-2174.1991 PMC257982

[65] Anand PK, Anand E, Bleck CK, Anes E, Griffiths G. Exosomal Hsp70 induces a pro-inflammatory response to foreign particles including mycobacteria. PLoS One. 2010;5(4):e10136 10.1371/journal.pone.0010136 20405033 PMC2853569

[66] Atay S, Gercel-Taylor C, Suttles J, Mor G, Taylor DD. Trophoblast-Derived Exosomes Mediate Monocyte Recruitment and Differentiation. American Journal of Reproductive Immunology. 2011;65(1):65–77. 10.1111/j.1600-0897.2010.00880.x 20560914

[67] Macdonald W. Selection of a strain of *Aedes-aegypti* susceptible to infectionwith semi-periodic *Brugia-malayi* . Annals of Tropical Medicine and Parasitology. 1962;56(3):368. 10.1080/00034983.1963.11686200 14101936

[68] Filipe V, Hawe A, Jiskoot W. Critical evaluation of Nanoparticle Tracking Analysis (NTA) by NanoSight for the measurement of nanoparticles and protein aggregates. Pharmaceutical research. 2010;27(5):796–810. 10.1007/s11095-010-0073-2 20204471 PMC2852530

[69] Gordon A, Hannon G. Fastx-toolkit. FASTQ/A short-reads pre-processing tools. 2010; Available: http://hannonlab.cshl.edu/fastx_toolkit

[70] Griffiths-Jones S, Saini HK, van Dongen S, Enright AJ. miRBase: tools for microRNA genomics. Nucleic Acids Research. 2008 1;36(Database issue):D154–8. 10.1093/nar/gkm952 17991681 PMC2238936

[71] Altschul SF, Gish W, Miller W, Myers EW, Lipman DJ. Basic local alignment search tool. Journal of Molecular Biology. 1990 10;215(3):403–410. 10.1016/S0022-2836(05)80360-2 2231712

[72] Wickham, H. ggplot2; 2009.

