# Supplementary figures and images for: Release of Small RNA-containing Exosome-like Vesicles from the Human Filarial Parasite Brugia malayi
Source: PLoS Negl Trop Dis. 2015 Sep 24;9(9):e0004069. doi: 10.1371/journal.pntd.0004069 (PMC4581865; doi:10.1371/journal.pntd.0004069)

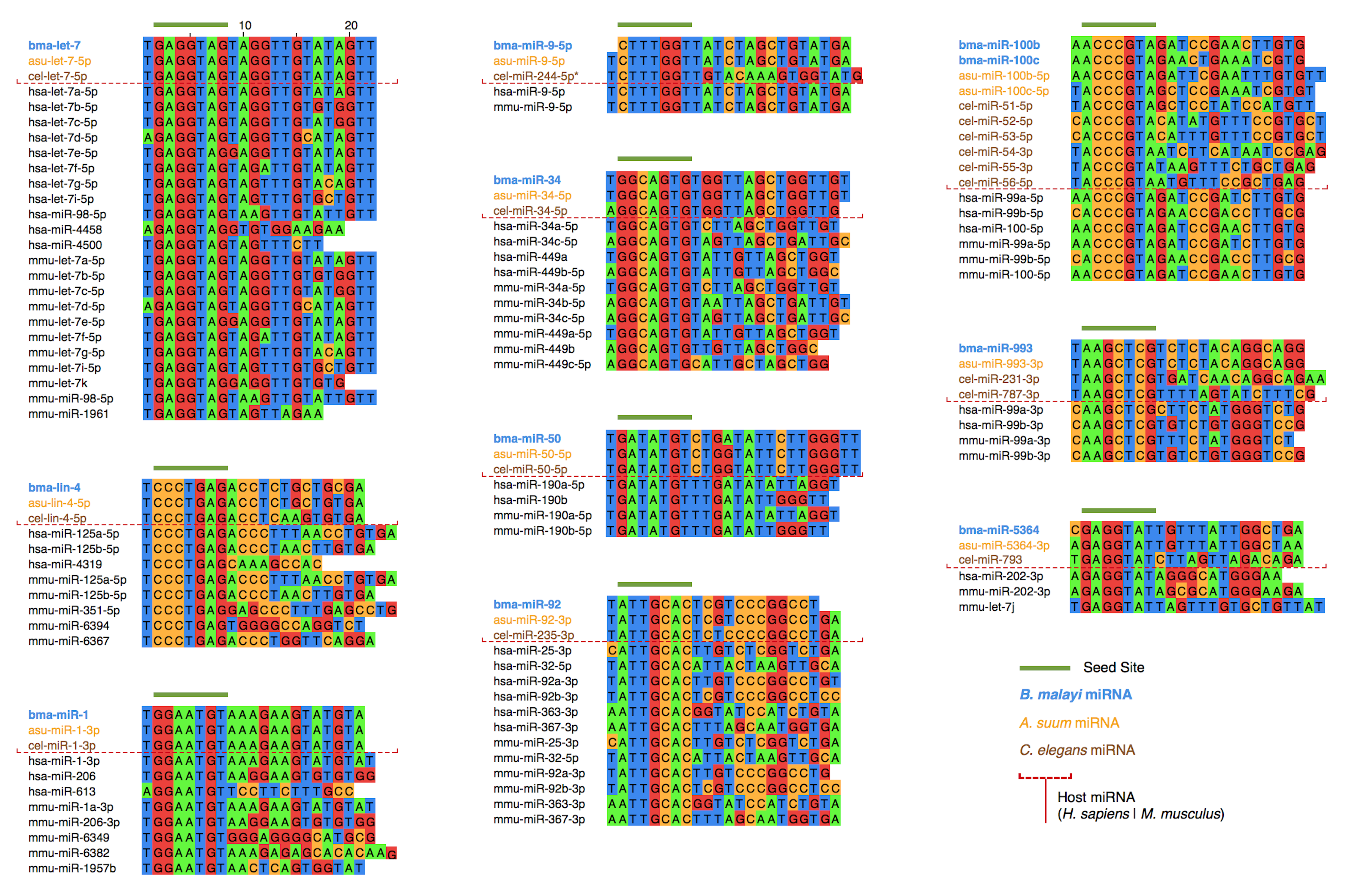

Supplement: S1 Fig — miRNAs are grouped by putative seed site and aligned. (TIFF) [file pntd.0004069.s003.tiff]

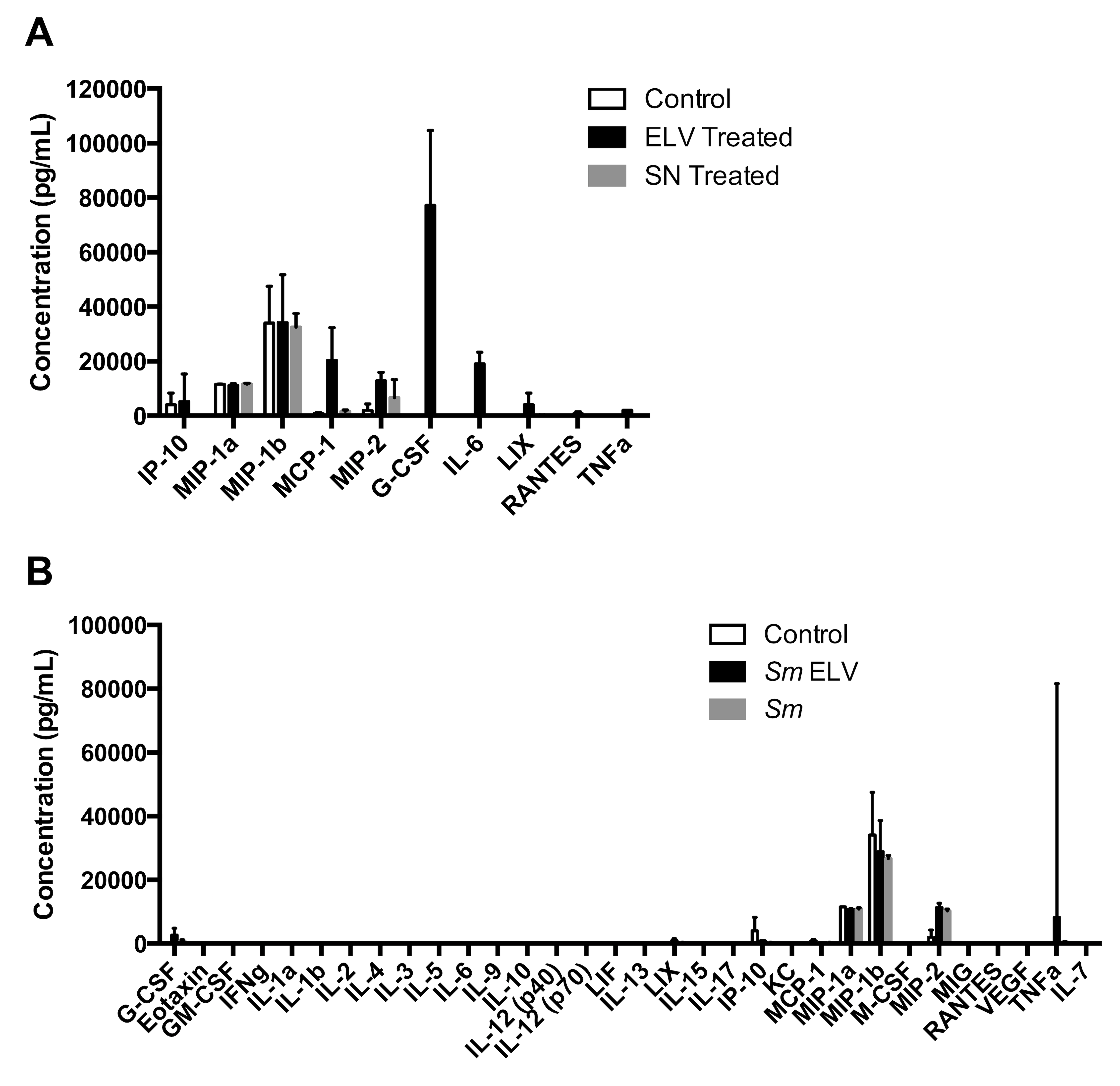

Supplement: S2 Fig — (A) J774A.1 macrophages (5 × 10^5^) were treated with approximately 4 × 10^8^ purified L3 stage ELVs, an equivalent volume of culture media supernatant from which ELVs had been depleted by centrifugation (SN Treated) or naïve culture media (control). The presence of 32 cytokines/chemokines was simultaneously assayed using the Milliplex MAP Mouse Cytokine/Chemokine kit (EDM Millipore) interfaced with a Bio-Plex System (Bio-Rad) utilizing Luminex xMAP technology (Luminex). The quantification of identified cytokines is presented. ELV treatment, but not the ELV depleted culture media, generates a classically activated phenotype. (B) J774A.1 macrophages were treated with approximately 4 × 10^8^ ELVs collected from a culture of Schistosoma mansoni invasive stage schistosomules as described for Brugia (Sm ELV), live S. mansoni schistosomules (300 per well; Sm) and naïve RPMI 1640 culture media (control). Macrophages were not activated by either schistosome preparation. (TIFF) [file pntd.0004069.s004.tiff]
